# Endogenous Honeybee Gut Microbiota Metabolize the Pesticide Clothianidin

**DOI:** 10.3390/microorganisms10030493

**Published:** 2022-02-23

**Authors:** Sarah El Khoury, Pierre Giovenazzo, Nicolas Derome

**Affiliations:** 1 Department of Biology, Laval University, Québec, QC G1V 0A6, Canada; sarah.el-khoury.1@ulaval.ca (S.E.K.); pierre.giovenazzo@bio.ulaval.ca (P.G.); 2 Institut de Biologie Intégrative et des Systèmes (IBIS), Laval University, Québec, QC G1V 0A6, Canada

**Keywords:** honeybee, clothianidin, microbiota, probiotic candidate, QuEChERS

## Abstract

Including probiotics in honeybee nutrition represents a promising solution for mitigating diseases, and recent evidence suggests that various microbes possess mechanisms that can bioremediate environmental pollutants. Thus, the use of probiotics capable of degrading pesticides used in modern agriculture would help to both reduce colony losses due to the exposure of foragers to these toxic molecules and improve honeybee health and wellbeing globally. We conducted in vitro experiments to isolate and identify probiotic candidates from bacterial isolates of the honeybee gut (i.e., endogenous strains) according to their ability to (i) grow in contact with three sublethal concentrations of the pesticide clothianidin (0.15, 1 and 10 ppb) and (ii) degrade clothianidin at 0.15 ppb. The isolated bacterial strains were indeed able to grow in contact with the three sublethal concentrations of clothianidin. Bacterial growth rate differed significantly depending on the probiotic candidate and the clothianidin concentration used. Clothianidin was degraded by seven endogenous honeybee gut bacteria, namely *Edwardsiella* sp., two *Serratia* sp., *Rahnella* sp., *Pantoea* sp., *Hafnia* sp. and *Enterobacter* sp., measured within 72 h under in vitro conditions. Our findings highlight that endogenous bacterial strains may constitute the base material from which to develop a promising probiotic strategy to mitigate the toxic effects of clothianidin exposure on honeybee colony health.

## 1. Introduction

The European honeybee (*Apis mellifera*) plays a crucial role in maintaining a functional and healthy ecological community through the pollination services it provides [1,2]. Furthermore, *Apis mellifera* is of major importance to many agricultural activities worldwide [3], considering that 75% of global food crop production relies on pollination services [4]. In recent decades, honeybee colonies have been impacted adversely, with mortalities reported around the world [5,6]. Various studies have highlighted multiple biotic [7,8] and abiotic stress factors [9,10] responsible for honeybee colony decline [1,3], which is particularly notable in regard to pollination services. Pesticide exposure is one factor that has impaired the ability of honeybees to pollinate efficiently [11,12,13].

Neonicotinoid pesticides began to be applied commonly in agricultural activities worldwide after the first neonicotinoid molecule was registered for use (i.e., imidacloprid) in 1991 [14], followed by nitenpyram and acetamiprid in 1995; thiamethoxam in 1998; thiacloprid in 2000; clothianidin in 2001; and dinotefuran in 2002 [15,16,17]. Initially, new era agricultural insect control pesticides, such as neonicotinoids, were projected to replace toxic agrochemicals, such as organophosphates, carbamates, pyrethroids, and other insecticides [14,18]. Unfortunately, at that time, their potential to negatively affect the environment and non-target organisms was underestimated [15,19]. After years of use, neonicotinoids have been found to be ubiquitous in the environment [20] and their persistence in soils [21] and water [22] has been shown to affect honeybees [23]. Acting as agonists of insect nicotinic acetylcholine receptors (nAChRs) [20], neonicotinoids cause neurological disorders in honeybees [24], other pollinators and many other non-target animals [25] by binding to the nicotinic receptors [26]. Many recent studies have confirmed the negative effect of neonicotinoids on *Apis mellifera* health, either in vivo [27,28,29,30] or in situ [10,31,32,33,34,35].

Honeybees possess few genes that can detoxify pesticides [36], although their social detoxification system [37] and collective immunity may compensate to a limited extent detoxifying gene repertoire [38]. Additionally, pesticide resistance was found in honeybees supplemented with a rich supply of pollen or nectar [35]. Furthermore, a study by Mao et al. (2013) [39] suggests that diet may improve pesticide resistance acquisition in honeybees by increasing detoxifying enzyme gene expression or physiological resilience. Interestingly, in recent decades, microbes have been shown to be able to degrade chemical compounds. This valuable ecosystemic service is referred to as microbial bioremediation [21,40,41,42]. Two types of microbes, bacteria (e.g., *Acinetobacter*, *Bacillus*, *Mycobacterium*, *Pseudomonas*, *Pseudoxanthomonas*, *Rhizobium*, *Rhodococcus*, *Sphingomonas*) and fungi (e.g., *Actinomycetes*, *Aspergillus*, *Fusarium*, and *Stenotrophomonas*) (reviewed in [20]), were shown to be able to degrade neonicotinoids. More specifically, studies reported degradation of clothianidin by microbes present in the surrounding environment, either bacteria (*Pseudomonas*) [21,43,44] or fungi (*Phanerochaete sordida*) [44]. Depending on their metabolisms, microorganisms degrade neonicotinoids into less or more toxic metabolites, thereby controlling their impact on environmental integrity [20]. However, the role of honeybee gut microbiota in the process of pesticide detoxification remains poorly understood [45].

The main goal of this research is to identify endogenous bacterial strains able to degrade the pesticide clothianidin, which is known to exert neurotoxic effects on honeybees [24,26,46]. As such, this is the first step in developing a probiotic strategy to mitigate the adverse effects of clothianidin exposure on honeybees. We hypothesize that endogenous honeybee gut bacterial strains could be able to degrade the clothianidin molecule into less harmful molecules, at least partially, thereby mitigating the pesticide’s adverse effects on honeybee physiology. To test our hypothesis, we assessed the potential of endogenous probiotic candidates to grow in contact with three sublethal concentrations of clothianidin (0.15, 1 and 10 ppb) under in vitro conditions and estimated the capacity of honeybee gut bacteria to degrade 0.15 ppb of clothianidin, a concentration level determined based on previous findings that a low dose of this pesticide (0.1 ppb) induces the most adverse impacts on honeybees [30].

## 2. Material and Methods

### 2.1. Isolation of Probiotic Candidates from Honeybee Gut Microbiota

**Figure 1:**
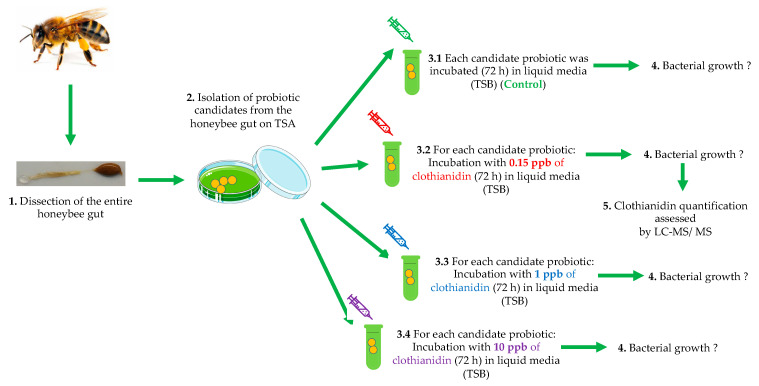
Experimental schema of the in vitro design (from left to right): (1) We started by isolating and dissecting 60 entire honeybee guts; we then (2) selected the bacterial probiotic candidates (PC) used in the study; (3) we incubated the isolated PC with (3.1) TSB media (control); and with three sublethal doses of clothianidin (0.15 ppb) (3.2); 1 ppb (3.3) and 10 ppb (3.4); (4) we monitored PC bacterial growth rate 72 h in all four conditions; and, finally, (5) we assessed clothianidin quantification with LC-MS/MS to measure degradation by the PC.

During the summer and winter of 2015 and 2016, probiotic candidates were isolated from the entire honeybee gut using the procedure described in detail in our previous study [47]. Honeybees (adults) for this study were sampled randomly on brood frames from European honeybee colonies (*Apis mellifera* L.) headed by queen sisters. Briefly, 60 healthy adult worker honeybees from five colonies were sampled from the livestock of the Centre de Recherche en Sciences Animales de Deschambault (CRSAD, Québec, QC, Canada). Individual guts were dissected under sterile conditions from the crop to the end of the rectum. The guts were then macerated, vortexed and placed into a sterile 10 mL Snap-cap tube (No. 62.554.002 PP*, Sarstedt Inc., Montréal, QC, Canada) (filled with 5 mL of PBS (1X) (Phosphate buffered saline). Serial dilutions (10^−^^1^ to 10^−^^4^) were prepared from the original vortexed PBS (1X) solution combined with honeybee gut tissue. A volume of 100 µL of each diluted solution was transferred to solid media of Tryptic Soy Agar (TSA) (Difco Inc., VWR, QC, Canada). TSA solid media was then incubated in a dark room at 37 °C for 48 h in aerobiosis. After two days of incubation and bacterial growth, we isolated each different colony from the solid TSA media. All distinct colony-forming units (CFU) were selected for transfer to new TSA media plates to obtain pure bacterial strains (i.e., probiotic candidates) (Figure 1). Probiotic candidates were stored in a 2 mL cryotube with glycerol (15% final concentration) at −80 °C for future use.

For probiotic candidate identification from DNA extraction, we followed the manufacturer’s protocol of the Dneasy Blood and Tissues kit (Qiagen Inc. (Toronto, ON, Canada), 2016; Pretreatment for Gram-Positive Bacteria In DNeasy R Blood & Tissue Handbook. 2006) using a resuspension of the cultured probiotic candidates in 200 µL of PBS 1X solution, followed by a thermal shock at 95 °C for 5 min. First, the 16S rRNA gene was targeted and amplified by PCR using the universal bacterial primers F-Tot (5′-GCAGGCCTAACACATGCAAGTC) and 1389R (5′-AGGCCCGGGAACGTATTCAC) (Sigma-Aldrich, Genosys). The PCR was conducted in a total volume of 50 µL: H_2_O 18 µL; TaKaRa (Taq) 25 µL, F-tot (10µM) 2.5 µL; 1389R (10 µM) 2.5 µL; and DNA 2 µL. After initial denaturation at 95 °C for 2 min, amplification was performed using 30 cycles of 1 min at 95 °C, 1 min at 55 °C and 1 min 30 s at 72 °C followed by a final extension at 72 °C for 5 min. Amplification products were run on 2.0% agarose gels and sent to the “Plate-forme d’Analyses Génomiques” of Laval University for Sanger DNA sequencing using universal primers. Taxonomic identification of 16S rRNA gene sequences was completed using the BLAST sequence analysis tool of the NCBI database “rRNA_typestrains/prokaryotic_16S_ribosomal_RNA”.

### 2.2. In Vitro Tests

#### 2.2.1. Growth of Honeybee Gut Probiotic Candidates

Probiotic candidates were cultured from glycerol stock stored at −80 °C, on solid media (TSA) in a dark room at 37 °C for 48 h to obtain a sufficient amount of bacterial CFU. Each probiotic candidate CFU was transferred into liquid culture media (TSB) and placed on a shaker (180 rpm) in a dark room at 37 °C for 48 h to obtain sufficient bacterial cells. The liquid culture of each probiotic candidate was calibrated with a spectrophotometer to a final concentration of 1 OD (Optical Density) at a wavelength of 600 nm. Then, 100 μL of the calibrated solution of each probiotic candidate was centrifuged and resuspended in 100 μL PBS (1X). A volume of 10 μL of each diluted bacterial culture was vortexed and transferred in triplicate to honeycomb microplate wells to achieve a final volume of 200 μL containing TSB (Tryptic Soy Broth) liquid culture media with 0.15, 1 and 10 ppb of clothianidin. Two controls (in triplicate) were used: controls containing liquid media (200 μL) and controls containing 10 μL of each bacterial strain (OD = 1) with 190 μL of TSB liquid media. Bacterial growth was measured using a Bioscreen-C plate reader (Growth Curves USA, Piscataway, NJ, USA). Microplates were incubated in darkness at 37 °C for 72 h. Every 15 min, OD was measured and recorded. All OD measurements were used to assess bacterial growth for each probiotic candidate. Bacterial growth graphs were generated and visualized using the package “ggplot2” with RStudio (Version 1.3.1093). For bacterial growth graphs, each dot represents the mean of OD (triplicate measures) observed every 6 h. To measure the effect of clothianidin dose on the growth of each bacterial strain, we tested the assumption of normality for each strain based on the OD measurements using a Shapiro–Wilk test [48] with RStudio. A repeated-measures ANOVA was used to test whether significant differences in the growth rate of probiotic candidates (PC) were observed under four different treatments (control; 0.15; 1 and 10 ppb) over time (significant *p*-values < 0.05). Multiple comparisons using an ANOVA analysis were computed between each different treatment (control; 0.15; 1 and 10 ppb) for each probiotic candidate at different time points (T6 h, T12 h, T18 h, T24 h, T30 h, T36 h, T42 h, T48 h, T54 h, T60 h, T66 h and T72 h) to detect significant differences (significant *p*-values < 0.05). *p*-values were adjusted with the Tukey’s method. Normality and homogeneity of variances were verified using the Shapiro test.

#### 2.2.2. Clothianidin Degradation by Honeybee Gut Probiotic Candidates

For quantification, clothianidin (CAS Number 210880-92-5, supplied by Sigma-Aldrich Inc., Ontario, Canada) was dissolved in distilled water to obtain three different stock solutions (0.15; 1 and 10 ppb (μg/L)). These three final concentrations were quantified by liquid chromatography-tandem mass spectrometry (LC-MS/MS) at the INRS (Institut National de la Recherche Scientifique, Québec, Canada) before use.

We used the QuEChERS method coupling chromatography and tandem mass spectrometry adapted from the study by Paradis et al. (2014) [49]. As a tool for calibration and recovery determination, methanol (MeOH) was used as stock standard and stored at 4 °C in a dark room. Atrazine-D5 was used as internal standard (IS). Both standards were purchased from CDN Isotopes (Pointe-Claire, Québec, QC, Canada).

We prepared (in triplicate) (i) bacterial culture tubes containing 15 mL of TSB liquid media adjusted with 0.15 ppb (clothianidin) (pesticide control) and (ii) bacterial culture tubes containing 15 mL of each bacterial culture in TSB liquid media, adjusted at OD = 1, with a clothianidin concentration of 0.15 ppb. Adjusted samples (probiotic candidates OD = 1 + clothianidin (0.15 ppb) and the pesticide control tubes) were incubated aerobically on a shaker (180 rpm) at 37 °C in a dark room. After T24, 48 and 72 h, we sampled 5 mL from each experimental culture tube (3 replicates per experimental group) that we placed on ice for transportation to the Institut National de la Recherche Scientifique (INRS, Québec Canada) for clothianidin quantification. Samples (5 mL) were mixed with acetonitrile (CH_3_CN—1.5 mL) and vortexed for 2 min. For extraction, the four following salts were added to each sample: magnesium sulfate (MgSO_4_—4 g), sodium chloride (NaCl—1 g), sodium citrate dihydrate (1 g) and disodium citrate sesquihydrate (0.5 g). The sample tubes were shaken up and down multiple times for 15 min, then centrifuged at room temperature for 5 min at 3000× *g*. After decanting, the supernatant (500 μL) was transferred to a new culture tube to dry in a nitrogen evaporator set at 40 °C. Once the pellet had dried, we added 100 μL of water:methanol (85:15) + atrazine-D5 (2 ppb) and transferred the contents to a new tube for clothianidin quantification using LC-MS/MS.

For LC-MS/MS instrumentation, liquid chromatography tests were performed using a Thermo Scientific TSQ QuantumTM Access MAX Triple Quadrupole Mass Spectrometer fitted with a Hypersil Gold aQ column (5 m, 2.1 × 100 mm) at T40 °C. To stop tailing peaks and boost peak shapes, column temperature and chromatographic gradient were optimized. To obtain optimal isolation of clothianidin molecules, a gradient method was used.

Chromatographic separation was carried out at 40 °C with a 10 μL injected volume and an 18 min run period. For 1 min, a mixture of 100% 10 mM acetate ammonium:methanol (85:15) was used as the mobile process. Flow rate was set to 0.25 mL/minutes. Clothianidin was isolated using the following elution protocol: linear gradient from 1.1 to 3 min (15% methanol: 85% 10 mM acetate ammonium) to (85% methanol: 15% 10 mM acetate ammonium) to return to starting conditions, 100% 10 mM acetate ammonium: methanol (85:15) was used for 10 min.

Clothianidin (*m*/*z* 250 (precursor ion); *m*/*z* 169 (product ion)); Atrazine-D5 (*m*/*z* 221 (precursor ion); *m*/*z* 179 (product ion)) was ionized by an electrospray source (ESI) in positive ionization in SRM mode, heated at 350 °C, and the ions were detected by triple quadrupole mass spectrometer (product ion). Xcalibur Software was used for quantification (Thermo Scientific, Québec, QC, Canada). The main parameter for separating, identifying, and quantifying specific compounds of interest from a complex solution is retention time (RT), which is one of the most significant chromatographic characteristics [50]. Extraction output was verified by looking at the signal areas of the IS that had been applied to the samples prior to extraction. The signal region of the IS (internal standard) applied to each sample before injection, as well as injections of quality control samples in each analytical run, were used to verify instrument results. The area ratio (peak area of clothianidin/peak area of internal norm) of each sample was compared to the calibration curve of the clothianidin standard for the quantitative analysis of clothianidin traces. The limit of detection of the LC-MS/MS was 0.05 ppb.

Significant differences in clothianidin (0.15 ppb) degradation by the seven probiotic candidates over time were calculated using a repeated-measures ANOVA (significant *p*-values < 0.05). Multiple comparisons using an ANOVA analysis were computed between (i) each pair of probiotic candidates and (ii) each probiotic candidate with the control (TSB + 0.15 ppb clothianidin) at different time points (T6 h, T12 h, T18 h, T24 h, T30 h, T36 h, T42 h, T48 h, T54 h, T60 h, T66 h and T72 h) to detect significant differences (significant *p*-values < 0.05). *p*-values were adjusted with Tukey’s method. Normality and homogeneity of variances were verified using the Shapiro test.

## 3. Results

### 3.1. Isolation of Probiotic Candidates from Honeybee Gut Microbiota

A total of seven endogenous honeybee gut bacterial strains were isolated on TSA solid media: *Edwardsiella* sp., two *Serratia* species (*Serratia* sp.1 and *Serratia* sp.2), *Rahnella* sp., *Pantoea* sp., *Hafnia* sp. and an *Enterobacter* sp. (Table 1).

### 3.2. In Vitro Tests

#### 3.2.1. Growth of Honeybee Gut Probiotic Candidates

Our results indicated a significant difference in probiotic candidate growth rate based on a double interaction with treatments (control; 0.15, 1 and 10 ppb) and over time (*p* = 0.0064) as well as a significant triple interaction between three experimental factors, “probiotic candidates”, “time” and “treatments” (*p* = 0.001) (Table 2). These results show that growth rates of various probiotic candidates differed significantly over time and between the different treatments.

**Figure 2:**
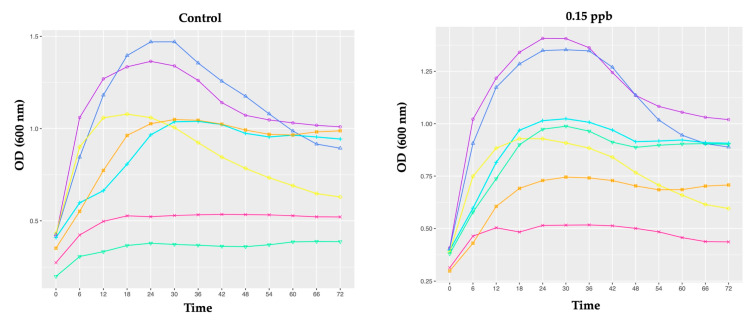

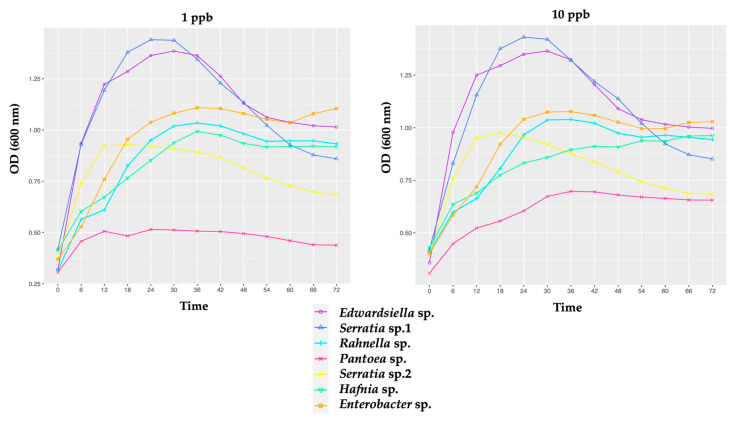
The bacterial growth curves represent the optical density (OD) units at 600 nm over time (hours). In order (from left to right, up to down), for *Edwardsiella* sp., *Serratia* sp.1, *Rahnella* sp., *Pantoea* sp., *Serratia* sp.2, *Hafnia* sp. and *Enterobacter* sp. cultured in TSB liquid media supplemented with 0 ppb (Control-first graph), 0.15 ppb (second graph), 1 ppb (third graph) and 10 ppb (fourth graph) for 72 h. The experiment was performed in triplicate. Each data point represents the average optical density (600 nm) measured every 6 h. Multiple comparisons using an ANOVA analysis were computed. *p*-values were adjusted with Tukey’s method (See Supplementary Material Table S2).

Our results show that all the honeybee gut probiotic candidates were able to grow in TSB culture media: (i) without clothianidin (control); and (ii) containing 0.15, 1 or 10 ppb of clothianidin. Probiotic candidate growth curves over time are shown in Figure 2.

First, we compared the impact of the different treatments (control; 0.15; 1 and 10 ppb) on the growth rate of each of the seven probiotic candidates.

**Figure 3:**
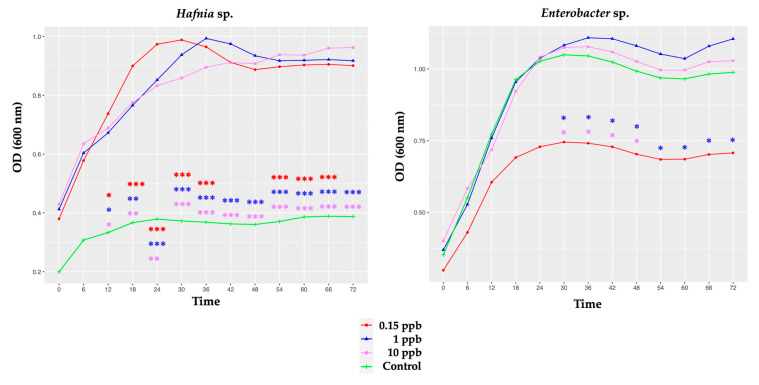
The bacterial growth curves represent the optical density (OD) units at 600 nm over time (hours) for *Hafnia* sp. and *Enterobacter* sp. cultured in TSB liquid media supplemented with 0 ppb (green curve) (unexposed group); 0.15 ppb (red curve); 1 ppb (blue curve) and 10 ppb (violet curve) for 72 h. Experiment was performed in triplicate. Each data point represents the average optical density (600 nm) measured every 6 h. Multiple comparisons using an ANOVA analysis were computed. *p*-values were adjusted with Tukey’s method.

The growth rate differed significantly between two probiotic candidates (*Hafnia* and *Enterobacter* sp.) exposed to the three clothianidin concentrations, as shown in Figure 3. The growth rates of the other probiotic candidates were similar under the same conditions (Supplementary Material Table S1).

For *Hafnia* sp., three clothianidin groups (0.15, 1 and 10 ppb) exhibited a significantly higher growth rate relative to the control, from T12 to the end of the experiment (Figure 3; Supplementary Material Table S1). Our observations suggest that clothianidin (three concentrations) induced a beneficial impact on the growth rate of *Hafnia* sp. In fact, clothianidin (three concentrations) exposure significantly doubled the bacterial optical density (OD) of *Hafnia* sp. relative to the control (Figure 3).

For *Enterobacter* sp., exposure to the lowest clothianidin concentration (0.15 ppb) significantly decreased the growth rate of *Enterobacter* sp. compared to the higher clothianidin doses (1 and 10 ppb), from T30 to T72 (Figure 3; Supplementary Material Table S1). Despite the significant differences observed between the three groups exposed to clothianidin, no significant difference was observed between the control (unexposed *Enterobacter* sp.) and the three groups exposed to *Enterobacter* sp. (0.15; 1 and 10 ppb) throughout the duration of the experiment (Supplementary Material Table S1).

For the five other probiotic candidates, *Edwardsiella* sp., *Serratia* sp.1, *Serratia* sp.2, *Rahnella* sp. and *Pantoea* sp., our results showed no significant difference between the four treatments (control; 0.15; 1 and 10 ppb) (Supplementary Material Table S2).

Second, by comparing the impact of the same treatment (control; 0.15; 1 or 10 ppb) on the growth rate of the seven probiotic candidates, we observed that the four treatments induced a significant difference in the comparative growth rate of all candidates (Figure 2; Supplementary Material Table S2).

At 0.15 ppb, comparisons between probiotic candidates’ growth rate at different specific time points support a significant greater impact (in decreasing order) on *Edwardsiella* sp., *Serratia* sp.1, *Rahnella* sp., *Hafnia* sp., *Enterobacter* sp. and *Pantoea* sp. (Figure 2; Supplementary Material Table S2). The growth rate of *Pantoea* sp. was the most negatively impacted, after 0.15 ppb of exposure throughout the experiment, relative to *Edwardsiella* sp., *Serratia* sp.1, *Rahnella* sp. and *Hafnia* sp.

At 1 ppb and 10 ppb, comparisons between the probiotic candidates’ growth rate at different specific time points support a significant greater impact (in decreasing order) on *Serratia* sp.1, *Edwardsiella* sp., *Enterobacter* sp., *Rahnella* sp., *Hafnia* sp. and *Pantoea* sp. (Figure 2; Supplementary Material Table S2).

At 1 ppb and 10 ppb, the growth rate of *Pantoea* sp. was also (as at 0.15 ppb) the most negatively impacted.

At 1 ppb and 10 ppb of clothianidin, we observed significant differences in *Pantoea* sp. relative to *Edwardsiella* sp., *Serratia* sp.1, *Hafnia* sp. and *Enterobacter* sp., while at 1 ppb we also observed a significant difference in *Pantoea* sp. relative to *Rahnella* sp. (Figure 2; Supplementary Material Table S2).

Interestingly, no significant difference was observed between *Serratia* sp.2 and the six other probiotic candidates (including *Serratia* sp.1), at 0.15; 1; and 10 ppb of clothianidin exposure and the unexposed experimental group (control) *(*Figure 2; Supplementary Material Table S2).

#### 3.2.2. Clothianidin Degradation by Honeybee Gut Probiotic Candidates

Our results indicated a significant difference in clothianidin degradation over time (*p* = 0.01) (Table 3). A fitted repeated-measures ANOVA model indicates the presence of a significant double interaction between “probiotic candidates” and “time” factors (*p* = 0.003) (Table 3). These results indicate that the degradation profile of clothianidin (0.15 ppb) over time differs between probiotic candidates.

The results of multiple comparisons indicate that the degradation rates of clothianidin by the seven probiotic candidates are equal to each other and to the control at T24 (Supplementary Material Table S3). However, at T48 and T72, clothianidin degradation profiles induced by the seven probiotic candidates are equal to each other, but they differ significantly from the control (Supplementary Material Tables S4 and S5). In fact, clothianidin (0.15 ppb) degradation is greater in the presence of probiotic candidates than without probiotic candidates. Regarding the degradation percentage of the clothianidin dose (0.15 ppb) after 48 h of incubation, three probiotic candidates (*Serratia* sp.1, *Rahnella* and *Edwardsiella* sp.) degraded the initial dose (0.15 ppb), while *Enterobacter* sp. degraded 68%, *Hafnia* sp. 81%, and *Serratia* sp.2 and *Pantoea* sp. 88% (Table 4) compared to the control. After 72 h of incubation, our results show 100% clothianidin degradation by the seven tested probiotic candidates (Table 4) relative to the control.

## 4. Discussion

In the present study, we observed how seven honeybee gut bacteria were able to (i) grow in contact with a concentration gradient of clothianidin (0.15, 1 and 10 ppb) and (ii) degrade the lower dose of clothianidin (0.15 ppb) completely within the same time frame.

### 4.1. Growth of Honeybee Gut Probiotic Candidates

First, the only probiotic candidate investigated in this study that showed a favorable response to clothianidin exposure (all three concentrations) was *Hafnia* sp. (Figure 3). Given that clothianidin exposure increased *Hafnia* sp. growth, our findings imply a non-toxic effect on the growth rate of *Hafnia* sp. and indicate a beneficial clothianidin dose–effect relationship with specific honeybee gut microbes in vitro.

Second, the other strains, *Enterobacter* sp., *Edwardsiella* sp., *Serratia* sp.1, *Serratia* sp.2, *Rahnella* sp. and *Pantoea* sp. (at three concentrations), grew as much as the unexposed candidate probiotic (Supplementary Material Table S1), suggesting a non-toxic clothianidin dose–effect with these honeybee gut microbes in vitro. Our findings may indicate microbial resistance to specific clothianidin concentrations.

Third, despite the nonsignificant difference observed between the unexposed and exposed bacterium, the growth rate of *Enterobacter* sp. was significantly reduced with 0.15 ppb clothianidin, compared to 1 and 10 ppb (Figure 3, Supplementary material Table S1). This significantly lower bacterial growth rate supports a toxic dose-effect relationship with clothianidin, suggesting a form of microbe tolerance in response to clothianidin toxicity in vitro. Tolerance defined by slow growth rates has been shown to be either inherited genetically [51], or nonhereditary, when microbe development is limited due to poor environmental conditions [51] and/or stressors exposure, for example, to an antibiotic [52] or a pesticide [53]. Our findings suggest that clothianidin-exposed *Enterobacter* sp. honeybee microbes did not acclimate to clothianidin exposure, instead of slowing their own development rate [53].

Fourth, in contrast, at both higher concentrations, *Enterobacter* sp. showed a better growth rate relative to 0.15 ppb (Figure 3), suggesting that *Enterobacter* sp. may also confer clothianidin resistance to honeybee gut microbes at higher doses. At 0.15 ppb, *Edwardsiella* sp., *Serratia* sp.1 and *Rahnella* sp. showed a greater growth rate relative to the other probiotic candidates (Figure 2; Supplementary Material Table S2) and degraded clothianidin completely after T48 (Table 4). Additionally, at higher concentrations (1 and 10 ppb), *Edwardsiella* sp. and *Serratia* sp.1 demonstrated an ability to achieve greater bacterial growth compared to the other probiotic candidates (Figure 2). Taken together, these results may suggest clothianidin resistance in these honeybee gut microbes. As previously demonstrated by [53], interactions between different pesticide concentrations (as 0.15, 1 and 10 ppb) and the metabolism of specific microbes may affect (i) the exposed candidate’s probiotic growth rate and (ii) pesticide degradation kinetics. The ability of several bacterial microbes to grow in contact with a xenobiotic was previously observed, including in neonicotinoid pesticides, such as thiamethoxam [54], imidacloprid [55] and clothianidin [43].

### 4.2. Clothianidin Degradation by Honeybee Gut Probiotic Candidates

After T48, three out of seven honeybee gut probiotic candidates (*Edwardsiella* sp., *Serratia* sp.1, and *Rahnella* sp.) cultures showed no evidence of clothianidin (0.15 ppb) (Table 4). The remaining four probiotic candidates (*Serratia* sp.2, *Pantoea* sp., *Hafnia* sp., and *Enterobacter* sp.) triggered partial clothianidin degradation at T48 (Table 4). However, at T72 of incubation, no traces of clothianidin were measured in contact with any of the seven probiotic candidates (Table 4), supporting complete bacterial degradation of clothianidin depending on some probiotic strains, as described previously [21,41]. This degradation may suggest that these microbes used clothianidin as a nutrient [56,57]. Due to the widespread accumulation of pesticides in the environment as a result of agricultural use, some microbes have developed genetic adaptation mechanisms to use these xenobiotic molecules as a food source, resulting in pesticide elimination [58]. The exploitation of pesticides by microbes as a source of nutrition may boost their development and survival [59,60], or may also negatively affect more sensitive species [57].

Previous studies have observed no [55], or partial, pesticide degradation [43,54], including partial degradation of clothianidin [21,43,44,61]. Studies have highlighted how pesticide degradation differs depending on the chemical agent involved [21,62], initial concentration used [43] or microbial strains involved [21,41]. While [43] and [55] did not observe neonicotinoid degradation at high concentrations with soil microbes, and honeybee gut microbes, respectively, the reverse was found by [54]. Mulligan and colleagues (2016), demonstrated the importance of a nutritive substrate and specific level of pesticide concentration for efficient degradation [43]. Regarding the clothianidin concentrations we used (0.15, 1 and 10 ppb), our results show a significant difference between bacterial growth rates in our tested probiotic candidates, depending on clothianidin concentration (Figure 3; Supplementary Material Tables S1 and S2), as in a previous study [57]. Additionally, anaerobic conditions and exposure to low temperatures (25 ± 2 °C) seem to accelerate clothianidin degradation in soil [43]. However, no difference was observed in clothianidin degradation under aerobic conditions at either tested temperature (25; 35 ± 2 °C). In the present study, an aerobic temperature (37 °C) may have impacted the partial clothianidin degradation generated by four out of seven probiotic candidates (*Serratia* sp.2, *Pantoea* sp., *Hafnia* sp. and *Enterobacter* sp.) after T48 of incubation (Table 4). It would be interesting to test the ability of different in vitro bacteria to degrade clothianidin more quickly.

To summarize, together, the above-mentioned case studies show how pesticide degradation efficiency depends on a variety of factors, among them microbial strain, nutrient media, chemical molecules characteristics and pesticide concentration used.

The absence of clothianidin (0.15 ppb) at T72 (Table 4) suggests that it was completely degraded and that all the candidates may have utilized the pesticide as a nutritional element. Alternatively, it is also possible that clothianidin was also degraded possibly in part by secondary metabolites produced by the probiotic candidates. As shown in a previous study, *Bombella apis* produced an antifungal secondary metabolite [63]. Moreover, other strains of *Rahnella* sp. (i.e., that completely degraded clothianidin molecules in our study) previously showed the ability to completely degrade profenofos (50 μg/mL), an organophosphate insecticide [64]. By showing how experimental conditions (such as nutrient media, pesticide concentration and specific microbial strain) condition the toxicity of a pesticide to microbes, our observations support the findings of [53].

The ability of the selected honeybee gut probiotic candidates to totally degrade clothianidin (Table 4) may confer clothianidin protection for the host. Based on our finding that exposure of honeybees to sublethal doses of clothianidin (0.1; 1 and 10 ppb) induced different levels of dysbiosis in honeybee gut microbiota [30], we hypothesized that clothianidin can be metabolized by some honeybee gut microbes. In contrast, Raymann et al. (2018) showed that honeybee environmental microbes totally digested imidacloprid, but did not indicate that honeybee gut strains could degrade it. This discrepancy may be attributable to our in vitro experimental design and the probiotic candidates we selected. Raymann et al. (2018) [55] used honeybee core microbes, *Snodgrassella alvi*, *Lactobacillus* Firm-4, *Lactobacillus* Firm-5, *Gilliamella apicola*, *Bifidobacterium*, *Bartonella apis* and *Frischella perrara*, whereas we used gut bacterial members not identified as being honeybee gut restricted (Table 1). Low abundance taxa have been shown to play an important role (i) in microbial population structure [65] and (ii) as keystone species in honeybee gut microbiota for maintaining the homeostasis of gut microbiota [30]. As mentioned above, by comparing our results with the observations of [55], we suggest that the honeybee gut microbiome environment may react differently to distinct neonicotinoid chemical compounds, considering also how the gut microbiome structure varies depending the pesticide type exposure [27,30,55].

As shown previously [2], two distinct strains of the same species do not necessarily respond in the same manner to the same pesticide chemical. In fact, the two bacterial strains *Serratia* sp.1 and *Serratia* sp.2 did not react in the same way, either in terms of their survival rate (Figure 2) or the time required to degrade clothianidin (0.15 ppb) (Table 4). These observations suggest (i) that strains belonging to the same bacterial genus may have different reactions in contact with exposure to the same chemical concentration, and (ii) how actual utilization of pesticides as a nutrient substrate may vary depending on the specific microbial strain.

Further in vitro analyses are needed to better understand (i) the ability of the tested probiotic candidates to degrade other neonicotinoid molecules and (ii) the ability of honeybee core members to degrade clothianidin molecules at sublethal doses (0.15, 1 and 10 ppb). Additionally, the in vitro conditions in this study must have had an impact on the clothianidin degradation rate. The capacity of microbes to degrade neonicotinoid pesticides differs depending on various factors, such as molecular structure [66], concentration [57], microbial metabolism [20,66,67], and environmental conditions: temperature, pH, experimental duration (i.e., number of days) and environmental oxygenation [20,43]. For example, the ability of *Pantoea agglomerans* and *Enterobacter* spp. to degrade another insecticide, the acephate molecule, was shown previously [56]. The authors demonstrated how these two bacteria benefit from acephate degradation, using it as a source of nutrients and energy. Moreover, in metabolizing acephate, the diamondback moth microbial gut community may be involved in developing insecticide resistance [56].

It would also be interesting to test to what extent probiotic candidates can degrade clothianidin at higher concentrations. Overall, our findings suggest that honeybee non-core members may be involved in generating microbiota-mediated clothianidin resistance in honeybees.

Overall, this research sheds light on how different microbial strains thrive and degrade clothianidin at sublethal concentrations. Our findings provide a novel perspective on the role of microorganisms in minimizing clothianidin’s negative influence on honeybee colony health. More in vivo experiments of probiotic candidates on clothianidin-exposed honeybees are required. For example, it would be fascinating to measure to what extent these probiotic candidates could mitigate the gut microbiota dysbiosis detected in prior investigations [30,68,69,70].

## Figures and Tables

**Table 1:** List of endogenous probiotic candidates isolated from the entire honeybee gut. Identity percentage (quantitative measure of two sequences’ similarity, indicating degree of relatedness) was identified with the BLAST database “rRNA_typestrains/prokaryotic_16S_ribosomal_RNA” from NCBI.

Sequence Name	Identity Percentage
*Edwardsiella tarda* strain ATCC 15947	99%
*Serratia marcescens* strain NBRC 102204	98%
*Rahnella woolbedingensis* strain FRB 227	99%
*Pantoea agglomerans* strain ATCC 27155	98%
*Serratia nematodiphila* strain DZ0503SBS1	98%
*Hafnia paralvei* strain ATCC 29927	99%
*Enterobacter* sp.	90%

**Table 2:** Repeated-Measures ANOVA results of probiotic candidate (PC) growth rates incubated with four different treatments (control; 0.15; 1 and 10 ppb) over time (significant *p*-values < 0.05).

	numDF	denDF	F-Value	*p*-Value
(Intercept)	1	672	44.59160	<0.0001
PC	6	56	0.27428	0.9467
Treatment	3	56	0.56574	0.6399
Time	12	672	136.42373	<0.0001
PC:Treatment	18	56	0.44272	0.9707
PC:Time	72	672	9.41233	<0.0001
Treatment:Time	36	672	1.71556	0.0064
PC:Treatment:Time	216	672	1.38909	0.0011

**Table 3:** Repeated-Measures ANOVA to test whether there are significant differences in clothianidin (0.15 ppb) degradation by the seven probiotic candidates (PC) over time (significant *p*-values < 0.05).

	numDF	denDF	F-Value	*p*-Value
(Intercept)	1	32	34.75724	<0.0001
PC	7	16	1.95330	0.1267
Time	2	32	4.88490	0.0141
PC:Time	14	32	3.18569	0.0033

**Table 4:** Degradation percentage of clothianidin dose by the seven probiotic candidates (PC) isolated from the honeybee gut assessed by LC-MS/MS at T24, T48 and T72. Each percentage of clothianidin degradation was evaluated based on comparison with the control solution without probiotics (TSB + clothianidin (0.15 ppb)) at T24, T48 and T72.

PC	T24	T48	T72
*Edwardsiella* sp.	61%	100% ***	100% ***
*Serratia* sp.1	1%	100% ***	100% ***
*Serratia* sp.2	48%	88% ***	100% ***
*Rahnella* sp.	61%	100% ***	100% ***
*Pantoea* sp.	48%	88% **	100% ***
*Hafnia* sp.	48%	81% **	100% ***
*Enterobacter* sp.	34%	68% **	100% ***
*vs*. TSB + clothianidin (0.15 ppb) (control)	0%	0%	0%

** 0.01; *** < 0.001 (See Supplementary Material Tables S4 and S5).

## Data Availability

Sequence data analyzed in the current study are available in the NCBI BioProject ID repository under the submission accession number SUB11001822.

## Supplementary Materials

The following supporting information can be downloaded at: https://www.mdpi.com/article/10.3390/microorganisms10030493/s1, Table S1: Significant difference in the bacterial growth rate for each probiotic candidate exposed to different treatments: 0.15; 1, 10 ppb and control (no pesticide) at specific time points. Significant *p*-values < 0.05; *p*-value adjustment with the Tukey method, Table S2: Significant difference in the bacterial growth rate between each pair of probiotic candidates exposed to different treatments: 0.15; 1, 10 ppb and control (no pesticide) at specific time points. Significant *p*-values < 0.05; *p*-value adjustment with the Tukey method, Table S3: Multiple comparisons of clothianidin degradation using an ANOVA analysis between each pair of probiotic candidates (PC) and with the control (TSB + 0.15 ppb Clothianidin) at T24, Table S4: Multiple comparisons of clothianidin degradation using an ANOVA analysis between each probiotic candidate with the control (TSB + 0.15 ppb Clothianidin) at T48, Table S5: Multiple comparisons of clothianidin degradation using an ANOVA analysis between each probiotic candidate with the control (TSB + 0.15 ppb Clothianidin) at T72.
