# Supplementary material for: Endogenous Honeybee Gut Microbiota Metabolize the Pesticide Clothianidin
Source: Microorganisms. 2022 Feb 23;10(3):493. doi: 10.3390/microorganisms10030493 (PMC8949661; doi:10.3390/microorganisms10030493)
Supplement: Supplementary file 1 [file microorganisms-10-00493-s001.zip › microorganisms-1573364-supplementary.pdf]

Endogenous honeybee gut microbiota metabolize the pesticide clothianidin

Sarah El Khoury [1,2], Pierre Giovenazzo [1] and Nicolas Derome [1,2].

[1] Department of Biology, Laval University, Québec, QC G1V 0A6, Canada
[2] Institut de Biologie Intégrative et des Systèmes (IBIS), Laval University, Québec, QC G1V 0A6, Canada

Contents

Table S1: Significant difference in the bacterial growth rate for each probiotic candidate exposed at different treatments: 0.15, 1, 10 ppb and control (no pesticide) at specific time points. Significant p-values < 0.05; p-value adjustment with Tukey's method. 2

Table S2: Significant difference in the bacterial growth rate between each pair of probiotic candidates exposed at different treatments: 0.15, 1, 10 ppb and control (no pesticide) at specific time points. Significant p-values < 0.05; p-value adjustment with Tukey's method. 15

Table S3: Multiple comparisons of clothianidin degradation using an ANOVA analysis between each pair of probiotic candidates (PC) and with the control (TSB + 0.15 ppb clothianidin) at T24. 37

Table S4: Multiple comparisons of clothianidin degradation using an ANOVA analysis between each probiotic candidate with the control (TSB + 0.15 ppb clothianidin) at T48. 38

Table S5: Multiple comparisons of clothianidin degradation using an ANOVA analysis between each probiotic candidate with the control (TSB + 0.15 ppb clothianidin) at T72. 38

Table S1: Significant difference in the bacterial growth rate for each probiotic candidate exposed at different treatments: 0.15, 1, 10 ppb and control (no pesticide) at specific time points. Significant p-values < 0.05; p-value adjustment with Tukey's method.

Probiotique = EDWARDSIELLA SP., Time = 0:					
contrast	estimate	SE	df	t.ratio	p.value
0.1 - 1	0.087627	0.0864	56	1.015	0.7417
0.1 - 10	0.050587	0.0864	56	0.586	0.9360
0.1 - B	-0.011213	0.0864	56	-0.130	0.9992
1 - 10	-0.037040	0.0864	56	-0.429	0.9733
1 - B	-0.098840	0.0864	56	-1.144	0.6639
10 - B	-0.061800	0.0864	56	-0.716	0.8905

Probiotique = SERRATIA SP.1, Time = 0:					
contrast	estimate	SE	df	t.ratio	p.value
0.1 - 1	-0.014773	0.1254	56	-0.118	0.9994
0.1 - 10	-0.016547	0.1254	56	-0.132	0.9992
0.1 - B	-0.022600	0.1254	56	-0.180	0.9979
1 - 10	-0.001773	0.1254	56	-0.014	1.0000
1 - B	-0.007827	0.1254	56	-0.062	0.9999
10 - B	-0.006053	0.1254	56	-0.048	1.0000

Probiotique = RAHNELLA SP. , Time = 0:					
contrast	estimate	SE	df	t.ratio	p.value
0.1 - 1	0.080760	0.1102	56	0.733	0.8835
0.1 - 10	-0.013840	0.1102	56	-0.126	0.9993
0.1 - B	-0.013347	0.1102	56	-0.121	0.9994
1 - 10	-0.094600	0.1102	56	-0.858	0.8261
1 - B	-0.094107	0.1102	56	-0.854	0.8284
10 - B	0.000493	0.1102	56	0.004	1.0000

Probiotique = PANTOEA SP., Time = 0:					
contrast	estimate	SE	df	t.ratio	p.value
0.1 - 1	0.008040	0.1367	56	0.059	0.9999
0.1 - 10	0.005480	0.1367	56	0.040	1.0000
0.1 - B	0.039393	0.1367	56	0.288	0.9916
1 - 10	-0.002560	0.1367	56	-0.019	1.0000
1 - B	0.031353	0.1367	56	0.229	0.9957
10 - B	0.033913	0.1367	56	0.248	0.9946

Probiotique = SERRATIA SP.2, Time = 0:					
contrast	estimate	SE	df	t.ratio	p.value
0.1 - 1	0.013533	0.2320	56	0.058	0.9999
0.1 - 10	-0.004680	0.2320	56	-0.020	1.0000
0.1 - B	-0.043407	0.2320	56	-0.187	0.9977
1 - 10	-0.018213	0.2320	56	-0.078	0.9998
1 - B	-0.056940	0.2320	56	-0.245	0.9947
10 - B	-0.038727	0.2320	56	-0.167	0.9983

Probiotique = HAFNIA SP., Time = 0:					
contrast	estimate	SE	df	t.ratio	p.value
0.1 - 1	-0.032907	0.1160	56	-0.284	0.9919
0.1 - 10	-0.049733	0.1160	56	-0.429	0.9733
0.1 - B	0.180213	0.1160	56	1.554	0.4128

1 - 10	-0.016827	0.1160	56	-0.145	0.9989
1 - B	0.213120	0.1160	56	1.838	0.2667
10 - B	0.229947	0.1160	56	1.983	0.2066

Probiotique = ENTEROBACTER SP., Time = 0:

contrast	estimate	SE	df	t.ratio	p.value
0.1 - 1	-0.071184	0.1243	56	-0.573	0.9398
0.1 - 10	-0.102624	0.1243	56	-0.826	0.8421
0.1 - B	-0.053824	0.1243	56	-0.433	0.9725
1 - 10	-0.031440	0.1243	56	-0.253	0.9943
1 - B	0.017360	0.1243	56	0.140	0.9990
10 - B	0.048800	0.1243	56	0.393	0.9793

Probiotique = EDWARDSIELLA SP., Time = 6:

contrast	estimate	SE	df	t.ratio	p.value
0.1 - 1	0.086222	0.0864	56	0.998	0.7510
0.1 - 10	0.043667	0.0864	56	0.506	0.9574
0.1 - B	-0.038278	0.0864	56	-0.443	0.9707
1 - 10	-0.042556	0.0864	56	-0.493	0.9604
1 - B	-0.124500	0.0864	56	-1.441	0.4794
10 - B	-0.081944	0.0864	56	-0.949	0.7787

Probiotique = SERRATIA SP.1, Time = 6:

contrast	estimate	SE	df	t.ratio	p.value
0.1 - 1	-0.024747	0.1254	56	-0.197	0.9972
0.1 - 10	0.074928	0.1254	56	0.597	0.9324
0.1 - B	0.060761	0.1254	56	0.484	0.9622
1 - 10	0.099674	0.1254	56	0.795	0.8565
1 - B	0.085508	0.1254	56	0.682	0.9036
10 - B	-0.014167	0.1254	56	-0.113	0.9995

Probiotique = RAHNELLA SP. , Time = 6:

contrast	estimate	SE	df	t.ratio	p.value
0.1 - 1	0.032986	0.1102	56	0.299	0.9906
0.1 - 10	-0.001056	0.1102	56	-0.010	1.0000
0.1 - B	-0.000694	0.1102	56	-0.006	1.0000
1 - 10	-0.034042	0.1102	56	-0.309	0.9897
1 - B	-0.033681	0.1102	56	-0.306	0.9900
10 - B	0.000361	0.1102	56	0.003	1.0000

Probiotique = PANTOEA SP., Time = 6:

contrast	estimate	SE	df	t.ratio	p.value
0.1 - 1	0.006811	0.1367	56	0.050	1.0000
0.1 - 10	0.015630	0.1367	56	0.114	0.9995
0.1 - B	0.040464	0.1367	56	0.296	0.9909
1 - 10	0.008819	0.1367	56	0.065	0.9999
1 - B	0.033653	0.1367	56	0.246	0.9947
10 - B	0.024833	0.1367	56	0.182	0.9978

Probiotique = SERRATIA SP.2, Time = 6:

contrast	estimate	SE	df	t.ratio	p.value
0.1 - 1	0.009153	0.2320	56	0.039	1.0000
0.1 - 10	-0.009375	0.2320	56	-0.040	1.0000
0.1 - B	-0.152056	0.2320	56	-0.655	0.9133
1 - 10	-0.018528	0.2320	56	-0.080	0.9998
1 - B	-0.161208	0.2320	56	-0.695	0.8987
10 - B	-0.142681	0.2320	56	-0.615	0.9269

Probiotique = HAFNIA SP., Time = 6:

contrast	estimate	SE	df	t.ratio	p.value
0.1 - 1	-0.025819	0.1160	56	-0.223	0.9961
0.1 - 10	-0.057264	0.1160	56	-0.494	0.9601

0.1 - B	0.270653	0.1160	56	2.334	0.1025
1 - 10	-0.031444	0.1160	56	-0.271	0.9929
1 - B	0.296472	0.1160	56	2.557	0.0621
10 - B	0.327917	0.1160	56	2.828	0.0320

Probiotique = ENTEROBACTER SP., Time = 6:

contrast	estimate	SE	df	t.ratio	p.value
0.1 - 1	-0.097653	0.1243	56	-0.786	0.8607
0.1 - 10	-0.153444	0.1243	56	-1.234	0.6079
0.1 - B	-0.121181	0.1243	56	-0.975	0.7642
1 - 10	-0.055792	0.1243	56	-0.449	0.9696
1 - B	-0.023528	0.1243	56	-0.189	0.9976
10 - B	0.032264	0.1243	56	0.260	0.9938

Probiotique = EDWARDSIELLA SP., Time = 12:

contrast	estimate	SE	df	t.ratio	p.value
0.1 - 1	-0.005236	0.0864	56	-0.061	0.9999
0.1 - 10	-0.032792	0.0864	56	-0.380	0.9812
0.1 - B	-0.052056	0.0864	56	-0.603	0.9308
1 - 10	-0.027556	0.0864	56	-0.319	0.9886
1 - B	-0.046819	0.0864	56	-0.542	0.9483
10 - B	-0.019264	0.0864	56	-0.223	0.9960

Probiotique = SERRATIA SP.1, Time = 12:

contrast	estimate	SE	df	t.ratio	p.value
0.1 - 1	-0.020506	0.1254	56	-0.164	0.9984
0.1 - 10	0.017452	0.1254	56	0.139	0.9990
0.1 - B	-0.008340	0.1254	56	-0.066	0.9999
1 - 10	0.037958	0.1254	56	0.303	0.9903
1 - B	0.012167	0.1254	56	0.097	0.9997
10 - B	-0.025792	0.1254	56	-0.206	0.9969

Probiotique = RAHNELLA SP. , Time = 12:

contrast	estimate	SE	df	t.ratio	p.value
0.1 - 1	0.204889	0.1102	56	1.859	0.2574
0.1 - 10	0.151944	0.1102	56	1.379	0.5179
0.1 - B	0.151944	0.1102	56	1.379	0.5179
1 - 10	-0.052944	0.1102	56	-0.480	0.9631
1 - B	-0.052944	0.1102	56	-0.480	0.9631
10 - B	0.000000	0.1102	56	0.000	1.0000

Probiotique = PANTOEA SP., Time = 12:

contrast	estimate	SE	df	t.ratio	p.value
0.1 - 1	-0.002270	0.1367	56	-0.017	1.0000
0.1 - 10	-0.019117	0.1367	56	-0.140	0.9990
0.1 - B	0.006813	0.1367	56	0.050	1.0000
1 - 10	-0.016847	0.1367	56	-0.123	0.9993
1 - B	0.009083	0.1367	56	0.066	0.9999
10 - B	0.025931	0.1367	56	0.190	0.9975

Probiotique = SERRATIA SP.2, Time = 12:

contrast	estimate	SE	df	t.ratio	p.value
0.1 - 1	-0.039139	0.2320	56	-0.169	0.9983
0.1 - 10	-0.066528	0.2320	56	-0.287	0.9917
0.1 - B	-0.175306	0.2320	56	-0.755	0.8739
1 - 10	-0.027389	0.2320	56	-0.118	0.9994
1 - B	-0.136167	0.2320	56	-0.587	0.9357
10 - B	-0.108778	0.2320	56	-0.469	0.9656

Probiotique = HAFNIA SP., Time = 12:

contrast	estimate	SE	df	t.ratio	p.value
0.1 - 1	0.065014	0.1160	56	0.561	0.9432

contrast	estimate	SE	df	t.ratio	p.value
0.1 - 10	0.048319	0.1160	56	0.417	0.9754
0.1 - B	0.404097	0.1160	56	3.485	0.0052
1 - 10	-0.016694	0.1160	56	-0.144	0.9989
1 - B	0.339083	0.1160	56	2.924	0.0249
10 - B	0.355778	0.1160	56	3.068	0.0170

Probiotique = ENTEROBACTER SP., Time = 12:

contrast	estimate	SE	df	t.ratio	p.value
0.1 - 1	-0.154097	0.1243	56	-1.240	0.6046
0.1 - 10	-0.112917	0.1243	56	-0.908	0.8004
0.1 - B	-0.167556	0.1243	56	-1.348	0.5369
1 - 10	0.041181	0.1243	56	0.331	0.9873
1 - B	-0.013458	0.1243	56	-0.108	0.9995
10 - B	-0.054639	0.1243	56	-0.440	0.9713

Probiotique = EDWARDSIELLA SP., Time = 18:

contrast	estimate	SE	df	t.ratio	p.value
0.1 - 1	0.055042	0.0864	56	0.637	0.9195
0.1 - 10	0.045889	0.0864	56	0.531	0.9511
0.1 - B	0.005972	0.0864	56	0.069	0.9999
1 - 10	-0.009153	0.0864	56	-0.106	0.9996
1 - B	-0.049069	0.0864	56	-0.568	0.9411
10 - B	-0.039917	0.0864	56	-0.462	0.9670

Probiotique = SERRATIA SP.1, Time = 18:

contrast	estimate	SE	df	t.ratio	p.value
0.1 - 1	-0.093577	0.1254	56	-0.746	0.8779
0.1 - 10	-0.090484	0.1254	56	-0.721	0.8881
0.1 - B	-0.110512	0.1254	56	-0.881	0.8146
1 - 10	0.003092	0.1254	56	0.025	1.0000
1 - B	-0.016935	0.1254	56	-0.135	0.9991
10 - B	-0.020028	0.1254	56	-0.160	0.9985

Probiotique = RAHNELLA SP. , Time = 18:

contrast	estimate	SE	df	t.ratio	p.value
0.1 - 1	0.143278	0.1102	56	1.300	0.5668
0.1 - 10	0.162069	0.1102	56	1.470	0.4619
0.1 - B	0.162069	0.1102	56	1.470	0.4619
1 - 10	0.018792	0.1102	56	0.170	0.9982
1 - B	0.018792	0.1102	56	0.170	0.9982
10 - B	0.000000	0.1102	56	0.000	1.0000

Probiotique = PANTOEA SP., Time = 18:

contrast	estimate	SE	df	t.ratio	p.value
0.1 - 1	0.000447	0.1367	56	0.003	1.0000
0.1 - 10	-0.073706	0.1367	56	-0.539	0.9490
0.1 - B	-0.043692	0.1367	56	-0.320	0.9886
1 - 10	-0.074153	0.1367	56	-0.543	0.9482
1 - B	-0.044139	0.1367	56	-0.323	0.9882
10 - B	0.030014	0.1367	56	0.220	0.9962

Probiotique = SERRATIA SP.2, Time = 18:

contrast	estimate	SE	df	t.ratio	p.value
0.1 - 1	-0.000847	0.2320	56	-0.004	1.0000
0.1 - 10	-0.046444	0.2320	56	-0.200	0.9971
0.1 - B	-0.149792	0.2320	56	-0.646	0.9167
1 - 10	-0.045597	0.2320	56	-0.196	0.9973
1 - B	-0.148944	0.2320	56	-0.642	0.9179
10 - B	-0.103347	0.2320	56	-0.445	0.9702

Probiotique = HAFNIA SP., Time = 18:

contrast	estimate	SE	df	t.ratio	p.value

contrast	estimate	SE	df	t.ratio	p.value
0.1 - 1	0.134014	0.1160	56	1.156	0.6569
0.1 - 10	0.124528	0.1160	56	1.074	0.7067
0.1 - B	0.533111	0.1160	56	4.598	0.0001
1 - 10	-0.009486	0.1160	56	-0.082	0.9998
1 - B	0.399097	0.1160	56	3.442	0.0059
10 - B	0.408583	0.1160	56	3.524	0.0046

Probiotique = ENTEROBACTER SP., Time = 18:

contrast	estimate	SE	df	t.ratio	p.value
0.1 - 1	-0.263375	0.1243	56	-2.119	0.1597
0.1 - 10	-0.230556	0.1243	56	-1.855	0.2592
0.1 - B	-0.271472	0.1243	56	-2.184	0.1403
1 - 10	0.032819	0.1243	56	0.264	0.9935
1 - B	-0.008097	0.1243	56	-0.065	0.9999
10 - B	-0.040917	0.1243	56	-0.329	0.9876

Probiotique = EDWARDSIELLA SP., Time = 24:

contrast	estimate	SE	df	t.ratio	p.value
0.1 - 1	0.043889	0.0864	56	0.508	0.9568
0.1 - 10	0.057806	0.0864	56	0.669	0.9082
0.1 - B	0.042722	0.0864	56	0.495	0.9600
1 - 10	0.013917	0.0864	56	0.161	0.9985
1 - B	-0.001167	0.0864	56	-0.014	1.0000
10 - B	-0.015083	0.0864	56	-0.175	0.9981

Probiotique = SERRATIA SP.1, Time = 24:

contrast	estimate	SE	df	t.ratio	p.value
0.1 - 1	-0.091096	0.1254	56	-0.726	0.8862
0.1 - 10	-0.081918	0.1254	56	-0.653	0.9140
0.1 - B	-0.120890	0.1254	56	-0.964	0.7704
1 - 10	0.009178	0.1254	56	0.073	0.9999
1 - B	-0.029794	0.1254	56	-0.238	0.9952
10 - B	-0.038972	0.1254	56	-0.311	0.9895

Probiotique = RAHNELLA SP. , Time = 24:

contrast	estimate	SE	df	t.ratio	p.value
0.1 - 1	0.064083	0.1102	56	0.581	0.9373
0.1 - 10	0.048389	0.1102	56	0.439	0.9714
0.1 - B	0.048389	0.1102	56	0.439	0.9714
1 - 10	-0.015694	0.1102	56	-0.142	0.9990
1 - B	-0.015694	0.1102	56	-0.142	0.9990
10 - B	0.000000	0.1102	56	0.000	1.0000

Probiotique = PANTOEA SP., Time = 24:

contrast	estimate	SE	df	t.ratio	p.value
0.1 - 1	0.000214	0.1367	56	0.002	1.0000
0.1 - 10	-0.090106	0.1367	56	-0.659	0.9118
0.1 - B	-0.007620	0.1367	56	-0.056	0.9999
1 - 10	-0.090319	0.1367	56	-0.661	0.9113
1 - B	-0.007833	0.1367	56	-0.057	0.9999
10 - B	0.082486	0.1367	56	0.604	0.9305

Probiotique = SERRATIA SP.2, Time = 24:

contrast	estimate	SE	df	t.ratio	p.value
0.1 - 1	0.007056	0.2320	56	0.030	1.0000
0.1 - 10	-0.027583	0.2320	56	-0.119	0.9994
0.1 - B	-0.131361	0.2320	56	-0.566	0.9417
1 - 10	-0.034639	0.2320	56	-0.149	0.9988
1 - B	-0.138417	0.2320	56	-0.596	0.9327
10 - B	-0.103778	0.2320	56	-0.447	0.9699

Probiotique = HAFNIA SP., Time = 24:

contrast	estimate	SE	df	t.ratio	p.value
0.1 - 1	0.121500	0.1160	56	1.048	0.7222
0.1 - 10	0.140875	0.1160	56	1.215	0.6201
0.1 - B	0.594847	0.1160	56	5.130	<.0001
1 - 10	0.019375	0.1160	56	0.167	0.9983
1 - B	0.473347	0.1160	56	4.082	0.0008
10 - B	0.453972	0.1160	56	3.915	0.0014

Probiotique = ENTEROBACTER SP., Time = 24:

contrast	estimate	SE	df	t.ratio	p.value
0.1 - 1	-0.308736	0.1243	56	-2.484	0.0735
0.1 - 10	-0.311764	0.1243	56	-2.508	0.0695
0.1 - B	-0.297944	0.1243	56	-2.397	0.0894
1 - 10	-0.003028	0.1243	56	-0.024	1.0000
1 - B	0.010792	0.1243	56	0.087	0.9998
10 - B	0.013819	0.1243	56	0.111	0.9995

Probiotique = EDWARDSIELLA SP., Time = 30:

contrast	estimate	SE	df	t.ratio	p.value
0.1 - 1	0.021375	0.0864	56	0.247	0.9946
0.1 - 10	0.041667	0.0864	56	0.482	0.9627
0.1 - B	0.066542	0.0864	56	0.770	0.8675
1 - 10	0.020292	0.0864	56	0.235	0.9954
1 - B	0.045167	0.0864	56	0.523	0.9532
10 - B	0.024875	0.0864	56	0.288	0.9916

Probiotique = SERRATIA SP.1, Time = 30:

contrast	estimate	SE	df	t.ratio	p.value
0.1 - 1	-0.084695	0.1254	56	-0.675	0.9060
0.1 - 10	-0.067462	0.1254	56	-0.538	0.9494
0.1 - B	-0.117240	0.1254	56	-0.935	0.7863
1 - 10	0.017233	0.1254	56	0.137	0.9991
1 - B	-0.032545	0.1254	56	-0.259	0.9938
10 - B	-0.049778	0.1254	56	-0.397	0.9786

Probiotique = RAHNELLA SP. , Time = 30:

contrast	estimate	SE	df	t.ratio	p.value
0.1 - 1	0.004667	0.1102	56	0.042	1.0000
0.1 - 10	-0.013264	0.1102	56	-0.120	0.9994
0.1 - B	-0.013264	0.1102	56	-0.120	0.9994
1 - 10	-0.017931	0.1102	56	-0.163	0.9984
1 - B	-0.017931	0.1102	56	-0.163	0.9984
10 - B	0.000000	0.1102	56	0.000	1.0000

Probiotique = PANTOEA SP., Time = 30:

contrast	estimate	SE	df	t.ratio	p.value
0.1 - 1	0.004081	0.1367	56	0.030	1.0000
0.1 - 10	-0.157391	0.1367	56	-1.152	0.6595
0.1 - B	-0.012613	0.1367	56	-0.092	0.9997
1 - 10	-0.161472	0.1367	56	-1.181	0.6410
1 - B	-0.016694	0.1367	56	-0.122	0.9993
10 - B	0.144778	0.1367	56	1.059	0.7154

Probiotique = SERRATIA SP.2, Time = 30:

contrast	estimate	SE	df	t.ratio	p.value
0.1 - 1	-0.000208	0.2320	56	-0.001	1.0000
0.1 - 10	-0.013278	0.2320	56	-0.057	0.9999
0.1 - B	-0.098194	0.2320	56	-0.423	0.9743
1 - 10	-0.013069	0.2320	56	-0.056	0.9999
1 - B	-0.097986	0.2320	56	-0.422	0.9744
10 - B	-0.084917	0.2320	56	-0.366	0.9831

Probiotique = HAFNIA SP., Time = 30:

contrast	estimate	SE	df	t.ratio	p.value
0.1 - 1	0.050653	0.1160	56	0.437	0.9718
0.1 - 10	0.129542	0.1160	56	1.117	0.6806
0.1 - B	0.615903	0.1160	56	5.312	<.0001
1 - 10	0.078889	0.1160	56	0.680	0.9041
1 - B	0.565250	0.1160	56	4.875	0.0001
10 - B	0.486361	0.1160	56	4.194	0.0006

Probiotique = ENTEROBACTER SP., Time = 30:

contrast	estimate	SE	df	t.ratio	p.value
0.1 - 1	-0.337153	0.1243	56	-2.712	0.0427
0.1 - 10	-0.329528	0.1243	56	-2.651	0.0496
0.1 - B	-0.303889	0.1243	56	-2.445	0.0803
1 - 10	0.007625	0.1243	56	0.061	0.9999
1 - B	0.033264	0.1243	56	0.268	0.9932
10 - B	0.025639	0.1243	56	0.206	0.9969

Probiotique = EDWARDSIELLA SP., Time = 36:

contrast	estimate	SE	df	t.ratio	p.value
0.1 - 1	-0.000181	0.0864	56	-0.002	1.0000
0.1 - 10	0.039958	0.0864	56	0.463	0.9669
0.1 - B	0.101208	0.0864	56	1.172	0.6470
1 - 10	0.040139	0.0864	56	0.465	0.9664
1 - B	0.101389	0.0864	56	1.174	0.6457
10 - B	0.061250	0.0864	56	0.709	0.8931

Probiotique = SERRATIA SP.1, Time = 36:

contrast	estimate	SE	df	t.ratio	p.value
0.1 - 1	0.002272	0.1254	56	0.018	1.0000
0.1 - 10	0.026637	0.1254	56	0.212	0.9966
0.1 - B	-0.007863	0.1254	56	-0.063	0.9999
1 - 10	0.024365	0.1254	56	0.194	0.9974
1 - B	-0.010135	0.1254	56	-0.081	0.9998
10 - B	-0.034500	0.1254	56	-0.275	0.9926

Probiotique = RAHNELLA SP. , Time = 36:

contrast	estimate	SE	df	t.ratio	p.value
0.1 - 1	-0.027500	0.1102	56	-0.250	0.9945
0.1 - 10	-0.032306	0.1102	56	-0.293	0.9911
0.1 - B	-0.032306	0.1102	56	-0.293	0.9911
1 - 10	-0.004806	0.1102	56	-0.044	1.0000
1 - B	-0.004806	0.1102	56	-0.044	1.0000
10 - B	0.000000	0.1102	56	0.000	1.0000

Probiotique = PANTOEA SP., Time = 36:

contrast	estimate	SE	df	t.ratio	p.value
0.1 - 1	0.010603	0.1367	56	0.078	0.9998
0.1 - 10	-0.180134	0.1367	56	-1.318	0.5556
0.1 - B	-0.015647	0.1367	56	-0.114	0.9995
1 - 10	-0.190736	0.1367	56	-1.396	0.5074
1 - B	-0.026250	0.1367	56	-0.192	0.9975
10 - B	0.164486	0.1367	56	1.203	0.6273

Probiotique = SERRATIA SP.2, Time = 36:

contrast	estimate	SE	df	t.ratio	p.value
0.1 - 1	-0.008236	0.2320	56	-0.035	1.0000
0.1 - 10	0.007208	0.2320	56	0.031	1.0000
0.1 - B	-0.040542	0.2320	56	-0.175	0.9981
1 - 10	0.015444	0.2320	56	0.067	0.9999
1 - B	-0.032306	0.2320	56	-0.139	0.9990

contrast	estimate	SE	df	t.ratio	p.value
10 - B	-0.047750	0.2320	56	-0.206	0.9969

Probiotique = HAFNIA SP., Time = 36:

contrast	estimate	SE	df	t.ratio	p.value
0.1 - 1	-0.028792	0.1160	56	-0.248	0.9946
0.1 - 10	0.069083	0.1160	56	0.596	0.9329
0.1 - B	0.596556	0.1160	56	5.145	<.0001
1 - 10	0.097875	0.1160	56	0.844	0.8332
1 - B	0.625347	0.1160	56	5.393	<.0001
10 - B	0.527472	0.1160	56	4.549	0.0002

Probiotique = ENTEROBACTER SP., Time = 36:

contrast	estimate	SE	df	t.ratio	p.value
0.1 - 1	-0.367264	0.1243	56	-2.955	0.0230
0.1 - 10	-0.335875	0.1243	56	-2.702	0.0438
0.1 - B	-0.303847	0.1243	56	-2.444	0.0804
1 - 10	0.031389	0.1243	56	0.253	0.9943
1 - B	0.063417	0.1243	56	0.510	0.9563
10 - B	0.032028	0.1243	56	0.258	0.9939

Probiotique = EDWARDSIELLA SP., Time = 42:

contrast	estimate	SE	df	t.ratio	p.value
0.1 - 1	-0.018222	0.0864	56	-0.211	0.9966
0.1 - 10	0.039528	0.0864	56	0.458	0.9679
0.1 - B	0.103347	0.0864	56	1.197	0.6316
1 - 10	0.057750	0.0864	56	0.669	0.9085
1 - B	0.121569	0.0864	56	1.407	0.5001
10 - B	0.063819	0.0864	56	0.739	0.8810

Probiotique = SERRATIA SP.1, Time = 42:

contrast	estimate	SE	df	t.ratio	p.value
0.1 - 1	0.041125	0.1254	56	0.328	0.9877
0.1 - 10	0.049792	0.1254	56	0.397	0.9786
0.1 - B	0.012264	0.1254	56	0.098	0.9997
1 - 10	0.008667	0.1254	56	0.069	0.9999
1 - B	-0.028861	0.1254	56	-0.230	0.9957
10 - B	-0.037528	0.1254	56	-0.299	0.9906

Probiotique = RAHNELLA SP. , Time = 42:

contrast	estimate	SE	df	t.ratio	p.value
0.1 - 1	-0.050056	0.1102	56	-0.454	0.9685
0.1 - 10	-0.052097	0.1102	56	-0.473	0.9648
0.1 - B	-0.052097	0.1102	56	-0.473	0.9648
1 - 10	-0.002042	0.1102	56	-0.019	1.0000
1 - B	-0.002042	0.1102	56	-0.019	1.0000
10 - B	0.000000	0.1102	56	0.000	1.0000

Probiotique = PANTOEA SP., Time = 42:

contrast	estimate	SE	df	t.ratio	p.value
0.1 - 1	0.008377	0.1367	56	0.061	0.9999
0.1 - 10	-0.181970	0.1367	56	-1.331	0.5472
0.1 - B	-0.021998	0.1367	56	-0.161	0.9985
1 - 10	-0.190347	0.1367	56	-1.393	0.5092
1 - B	-0.030375	0.1367	56	-0.222	0.9961
10 - B	0.159972	0.1367	56	1.170	0.6478

Probiotique = SERRATIA SP.2, Time = 42:

contrast	estimate	SE	df	t.ratio	p.value
0.1 - 1	-0.026611	0.2320	56	-0.115	0.9995
0.1 - 10	0.003153	0.2320	56	0.014	1.0000
0.1 - B	-0.004889	0.2320	56	-0.021	1.0000
1 - 10	0.029764	0.2320	56	0.128	0.9992

contrast	estimate	SE	df	t.ratio	p.value
1 - B	0.021722	0.2320	56	0.094	0.9997
10 - B	-0.008042	0.2320	56	-0.035	1.0000

Probiotique = HAFNIA SP., Time = 42:

contrast	estimate	SE	df	t.ratio	p.value
0.1 - 1	-0.062917	0.1160	56	-0.543	0.9482
0.1 - 10	0.000333	0.1160	56	0.003	1.0000
0.1 - B	0.549333	0.1160	56	4.737	0.0001
1 - 10	0.063250	0.1160	56	0.545	0.9474
1 - B	0.612250	0.1160	56	5.280	<.0001
10 - B	0.549000	0.1160	56	4.735	0.0001

Probiotique = ENTEROBACTER SP., Time = 42:

contrast	estimate	SE	df	t.ratio	p.value
0.1 - 1	-0.376542	0.1243	56	-3.029	0.0189
0.1 - 10	-0.330458	0.1243	56	-2.658	0.0487
0.1 - B	-0.295361	0.1243	56	-2.376	0.0936
1 - 10	0.046083	0.1243	56	0.371	0.9824
1 - B	0.081181	0.1243	56	0.653	0.9140
10 - B	0.035097	0.1243	56	0.282	0.9921

Probiotique = EDWARDSIELLA SP., Time = 48:

contrast	estimate	SE	df	t.ratio	p.value
0.1 - 1	0.005319	0.0864	56	0.062	0.9999
0.1 - 10	0.043931	0.0864	56	0.509	0.9567
0.1 - B	0.063097	0.0864	56	0.731	0.8844
1 - 10	0.038611	0.0864	56	0.447	0.9699
1 - B	0.057778	0.0864	56	0.669	0.9083
10 - B	0.019167	0.0864	56	0.222	0.9961

Probiotique = SERRATIA SP.1, Time = 48:

contrast	estimate	SE	df	t.ratio	p.value
0.1 - 1	0.001911	0.1254	56	0.015	1.0000
0.1 - 10	-0.001716	0.1254	56	-0.014	1.0000
0.1 - B	-0.039355	0.1254	56	-0.314	0.9892
1 - 10	-0.003628	0.1254	56	-0.029	1.0000
1 - B	-0.041267	0.1254	56	-0.329	0.9876
10 - B	-0.037639	0.1254	56	-0.300	0.9905

Probiotique = RAHNELLA SP. , Time = 48:

contrast	estimate	SE	df	t.ratio	p.value
0.1 - 1	-0.068069	0.1102	56	-0.618	0.9260
0.1 - 10	-0.060125	0.1102	56	-0.546	0.9474
0.1 - B	-0.060125	0.1102	56	-0.546	0.9474
1 - 10	0.007944	0.1102	56	0.072	0.9999
1 - B	0.007944	0.1102	56	0.072	0.9999
10 - B	0.000000	0.1102	56	0.000	1.0000

Probiotique = PANTOEA SP., Time = 48:

contrast	estimate	SE	df	t.ratio	p.value
0.1 - 1	0.006071	0.1367	56	0.044	1.0000
0.1 - 10	-0.179693	0.1367	56	-1.315	0.5576
0.1 - B	-0.033138	0.1367	56	-0.242	0.9949
1 - 10	-0.185764	0.1367	56	-1.359	0.5299
1 - B	-0.039208	0.1367	56	-0.287	0.9917
10 - B	0.146556	0.1367	56	1.072	0.7077

Probiotique = SERRATIA SP.2, Time = 48:

contrast	estimate	SE	df	t.ratio	p.value
0.1 - 1	-0.049250	0.2320	56	-0.212	0.9966
0.1 - 10	-0.024056	0.2320	56	-0.104	0.9996
0.1 - B	-0.017514	0.2320	56	-0.075	0.9998
1 - 10	0.025194	0.2320	56	0.109	0.9995

1 - B	0.031736	0.2320	56	0.137	0.9991
10 - B	0.006542	0.2320	56	0.028	1.0000

Probiotique = HAFNIA SP., Time = 48:

contrast	estimate	SE	df	t.ratio	p.value
0.1 - 1	-0.047347	0.1160	56	-0.408	0.9768
0.1 - 10	-0.020694	0.1160	56	-0.178	0.9980
0.1 - B	0.526750	0.1160	56	4.543	0.0002
1 - 10	0.026653	0.1160	56	0.230	0.9957
1 - B	0.574097	0.1160	56	4.951	<.0001
10 - B	0.547444	0.1160	56	4.721	0.0001

Probiotique = ENTEROBACTER SP., Time = 48:

contrast	estimate	SE	df	t.ratio	p.value
0.1 - 1	-0.377417	0.1243	56	-3.036	0.0185
0.1 - 10	-0.323264	0.1243	56	-2.601	0.0560
0.1 - B	-0.289431	0.1243	56	-2.328	0.1038
1 - 10	0.054153	0.1243	56	0.436	0.9721
1 - B	0.087986	0.1243	56	0.708	0.8936
10 - B	0.033833	0.1243	56	0.272	0.9929

Probiotique = EDWARDSIELLA SP., Time = 54:

contrast	estimate	SE	df	t.ratio	p.value
0.1 - 1	0.020667	0.0864	56	0.239	0.9951
0.1 - 10	0.044250	0.0864	56	0.512	0.9558
0.1 - B	0.035333	0.0864	56	0.409	0.9767
1 - 10	0.023583	0.0864	56	0.273	0.9928
1 - B	0.014667	0.0864	56	0.170	0.9982
10 - B	-0.008917	0.0864	56	-0.103	0.9996

Probiotique = SERRATIA SP.1, Time = 54:

contrast	estimate	SE	df	t.ratio	p.value
0.1 - 1	-0.005293	0.1254	56	-0.042	1.0000
0.1 - 10	-0.002795	0.1254	56	-0.022	1.0000
0.1 - B	-0.062226	0.1254	56	-0.496	0.9596
1 - 10	0.002498	0.1254	56	0.020	1.0000
1 - B	-0.056933	0.1254	56	-0.454	0.9686
10 - B	-0.059431	0.1254	56	-0.474	0.9645

Probiotique = RAHNELLA SP. , Time = 54:

contrast	estimate	SE	df	t.ratio	p.value
0.1 - 1	-0.026958	0.1102	56	-0.245	0.9948
0.1 - 10	-0.037222	0.1102	56	-0.338	0.9866
0.1 - B	-0.037222	0.1102	56	-0.338	0.9866
1 - 10	-0.010264	0.1102	56	-0.093	0.9997
1 - B	-0.010264	0.1102	56	-0.093	0.9997
10 - B	0.000000	0.1102	56	0.000	1.0000

Probiotique = PANTOEA SP., Time = 54:

contrast	estimate	SE	df	t.ratio	p.value
0.1 - 1	0.003255	0.1367	56	0.024	1.0000
0.1 - 10	-0.186175	0.1367	56	-1.362	0.5280
0.1 - B	-0.048231	0.1367	56	-0.353	0.9848
1 - 10	-0.189431	0.1367	56	-1.386	0.5133
1 - B	-0.051486	0.1367	56	-0.377	0.9816
10 - B	0.137944	0.1367	56	1.009	0.7447

Probiotique = SERRATIA SP.2, Time = 54:

contrast	estimate	SE	df	t.ratio	p.value
0.1 - 1	-0.058653	0.2320	56	-0.253	0.9943
0.1 - 10	-0.036986	0.2320	56	-0.159	0.9985
0.1 - B	-0.026722	0.2320	56	-0.115	0.9994
1 - 10	0.021667	0.2320	56	0.093	0.9997

contrast	estimate	SE	df	t.ratio	p.value
1 - B	0.031931	0.2320	56	0.138	0.9991
10 - B	0.010264	0.2320	56	0.044	1.0000

Probiotique = HAFNIA SP., Time = 54:

contrast	estimate	SE	df	t.ratio	p.value
0.1 - 1	-0.020014	0.1160	56	-0.173	0.9982
0.1 - 10	-0.040653	0.1160	56	-0.351	0.9851
0.1 - B	0.526653	0.1160	56	4.542	0.0002
1 - 10	-0.020639	0.1160	56	-0.178	0.9980
1 - B	0.546667	0.1160	56	4.714	0.0001
10 - B	0.567306	0.1160	56	4.892	0.0001

Probiotique = ENTEROBACTER SP., Time = 54:

contrast	estimate	SE	df	t.ratio	p.value
0.1 - 1	-0.367000	0.1243	56	-2.952	0.0232
0.1 - 10	-0.311681	0.1243	56	-2.507	0.0697
0.1 - B	-0.284069	0.1243	56	-2.285	0.1138
1 - 10	0.055319	0.1243	56	0.445	0.9703
1 - B	0.082931	0.1243	56	0.667	0.9090
10 - B	0.027611	0.1243	56	0.222	0.9961

Probiotique = EDWARDSIELLA SP., Time = 60:

contrast	estimate	SE	df	t.ratio	p.value
0.1 - 1	0.017764	0.0864	56	0.206	0.9969
0.1 - 10	0.038333	0.0864	56	0.444	0.9705
0.1 - B	0.023736	0.0864	56	0.275	0.9927
1 - 10	0.020569	0.0864	56	0.238	0.9952
1 - B	0.005972	0.0864	56	0.069	0.9999
10 - B	-0.014597	0.0864	56	-0.169	0.9983

Probiotique = SERRATIA SP.1, Time = 60:

contrast	estimate	SE	df	t.ratio	p.value
0.1 - 1	0.017821	0.1254	56	0.142	0.9990
0.1 - 10	0.021670	0.1254	56	0.173	0.9981
0.1 - B	-0.041608	0.1254	56	-0.332	0.9873
1 - 10	0.003849	0.1254	56	0.031	1.0000
1 - B	-0.059429	0.1254	56	-0.474	0.9645
10 - B	-0.063278	0.1254	56	-0.505	0.9577

Probiotique = RAHNELLA SP. , Time = 60:

contrast	estimate	SE	df	t.ratio	p.value
0.1 - 1	-0.024681	0.1102	56	-0.224	0.9960
0.1 - 10	-0.042181	0.1102	56	-0.383	0.9807
0.1 - B	-0.042181	0.1102	56	-0.383	0.9807
1 - 10	-0.017500	0.1102	56	-0.159	0.9986
1 - B	-0.017500	0.1102	56	-0.159	0.9986
10 - B	0.000000	0.1102	56	0.000	1.0000

Probiotique = PANTOEA SP., Time = 60:

contrast	estimate	SE	df	t.ratio	p.value
0.1 - 1	-0.003190	0.1367	56	-0.023	1.0000
0.1 - 10	-0.207259	0.1367	56	-1.516	0.4347
0.1 - B	-0.070648	0.1367	56	-0.517	0.9547
1 - 10	-0.204069	0.1367	56	-1.493	0.4484
1 - B	-0.067458	0.1367	56	-0.494	0.9602
10 - B	0.136611	0.1367	56	1.000	0.7503

Probiotique = SERRATIA SP.2, Time = 60:

contrast	estimate	SE	df	t.ratio	p.value
0.1 - 1	-0.068208	0.2320	56	-0.294	0.9911
0.1 - 10	-0.053208	0.2320	56	-0.229	0.9957
0.1 - B	-0.031319	0.2320	56	-0.135	0.9991
1 - 10	0.015000	0.2320	56	0.065	0.9999

1 - B	0.036889	0.2320	56	0.159	0.9986
10 - B	0.021889	0.2320	56	0.094	0.9997

Probiotique = HAFNIA SP., Time = 60:

contrast	estimate	SE	df	t.ratio	p.value
0.1 - 1	-0.016083	0.1160	56	-0.139	0.9990
0.1 - 10	-0.033333	0.1160	56	-0.287	0.9916
0.1 - B	0.517042	0.1160	56	4.459	0.0002
1 - 10	-0.017250	0.1160	56	-0.149	0.9988
1 - B	0.533125	0.1160	56	4.598	0.0001
10 - B	0.550375	0.1160	56	4.746	0.0001

Probiotique = ENTEROBACTER SP., Time = 60:

contrast	estimate	SE	df	t.ratio	p.value
0.1 - 1	-0.350514	0.1243	56	-2.820	0.0326
0.1 - 10	-0.310889	0.1243	56	-2.501	0.0707
0.1 - B	-0.279847	0.1243	56	-2.251	0.1222
1 - 10	0.039625	0.1243	56	0.319	0.9887
1 - B	0.070667	0.1243	56	0.568	0.9410
10 - B	0.031042	0.1243	56	0.250	0.9945

Probiotique = EDWARDSIELLA SP., Time = 66:

contrast	estimate	SE	df	t.ratio	p.value
0.1 - 1	0.009306	0.0864	56	0.108	0.9995
0.1 - 10	0.027444	0.0864	56	0.318	0.9888
0.1 - B	0.012833	0.0864	56	0.149	0.9988
1 - 10	0.018139	0.0864	56	0.210	0.9967
1 - B	0.003528	0.0864	56	0.041	1.0000
10 - B	-0.014611	0.0864	56	-0.169	0.9983

Probiotique = SERRATIA SP.1, Time = 66:

contrast	estimate	SE	df	t.ratio	p.value
0.1 - 1	0.026686	0.1254	56	0.213	0.9966
0.1 - 10	0.033355	0.1254	56	0.266	0.9933
0.1 - B	-0.010854	0.1254	56	-0.087	0.9998
1 - 10	0.006669	0.1254	56	0.053	0.9999
1 - B	-0.037540	0.1254	56	-0.299	0.9906
10 - B	-0.044208	0.1254	56	-0.352	0.9848

Probiotique = RAHNELLA SP. , Time = 66:

contrast	estimate	SE	df	t.ratio	p.value
0.1 - 1	-0.037500	0.1102	56	-0.340	0.9863
0.1 - 10	-0.044819	0.1102	56	-0.407	0.9771
0.1 - B	-0.044819	0.1102	56	-0.407	0.9771
1 - 10	-0.007319	0.1102	56	-0.066	0.9999
1 - B	-0.007319	0.1102	56	-0.066	0.9999
10 - B	0.000000	0.1102	56	0.000	1.0000

Probiotique = PANTOEA SP., Time = 66:

contrast	estimate	SE	df	t.ratio	p.value
0.1 - 1	-0.002249	0.1367	56	-0.016	1.0000
0.1 - 10	-0.218721	0.1367	56	-1.600	0.3868
0.1 - B	-0.083568	0.1367	56	-0.611	0.9280
1 - 10	-0.216472	0.1367	56	-1.584	0.3960
1 - B	-0.081319	0.1367	56	-0.595	0.9332
10 - B	0.135153	0.1367	56	0.989	0.7564

Probiotique = SERRATIA SP.2, Time = 66:

contrast	estimate	SE	df	t.ratio	p.value
0.1 - 1	-0.083028	0.2320	56	-0.358	0.9841
0.1 - 10	-0.074667	0.2320	56	-0.322	0.9884
0.1 - B	-0.032944	0.2320	56	-0.142	0.9990

contrast	estimate	SE	df	t.ratio	p.value
1 - 10	0.008361	0.2320	56	0.036	1.0000
1 - B	0.050083	0.2320	56	0.216	0.9964
10 - B	0.041722	0.2320	56	0.180	0.9979

Probiotique = HAFNIA SP., Time = 66:

contrast	estimate	SE	df	t.ratio	p.value
0.1 - 1	-0.016361	0.1160	56	-0.141	0.9990
0.1 - 10	-0.055111	0.1160	56	-0.475	0.9642
0.1 - B	0.516708	0.1160	56	4.456	0.0002
1 - 10	-0.038750	0.1160	56	-0.334	0.9870
1 - B	0.533069	0.1160	56	4.597	0.0001
10 - B	0.571819	0.1160	56	4.931	<.0001

Probiotique = ENTEROBACTER SP., Time = 66:

contrast	estimate	SE	df	t.ratio	p.value
0.1 - 1	-0.377139	0.1243	56	-3.034	0.0186
0.1 - 10	-0.322806	0.1243	56	-2.597	0.0565
0.1 - B	-0.280153	0.1243	56	-2.254	0.1215
1 - 10	0.054333	0.1243	56	0.437	0.9718
1 - B	0.096986	0.1243	56	0.780	0.8631
10 - B	0.042653	0.1243	56	0.343	0.9860

Probiotique = EDWARDSIELLA SP., Time = 72:

contrast	estimate	SE	df	t.ratio	p.value
0.1 - 1	0.005333	0.0864	56	0.062	0.9999
0.1 - 10	0.022333	0.0864	56	0.259	0.9939
0.1 - B	0.010000	0.0864	56	0.116	0.9994
1 - 10	0.017000	0.0864	56	0.197	0.9973
1 - B	0.004667	0.0864	56	0.054	0.9999
10 - B	-0.012333	0.0864	56	-0.143	0.9989

Probiotique = SERRATIA SP.1, Time = 72:

contrast	estimate	SE	df	t.ratio	p.value
0.1 - 1	0.029000	0.1254	56	0.231	0.9956
0.1 - 10	0.037333	0.1254	56	0.298	0.9907
0.1 - B	-0.005000	0.1254	56	-0.040	1.0000
1 - 10	0.008333	0.1254	56	0.066	0.9999
1 - B	-0.034000	0.1254	56	-0.271	0.9930
10 - B	-0.042333	0.1254	56	-0.338	0.9866

Probiotique = RAHNELLA SP. , Time = 72:

contrast	estimate	SE	df	t.ratio	p.value
0.1 - 1	-0.025333	0.1102	56	-0.230	0.9957
0.1 - 10	-0.036333	0.1102	56	-0.330	0.9875
0.1 - B	-0.036333	0.1102	56	-0.330	0.9875
1 - 10	-0.011000	0.1102	56	-0.100	0.9996
1 - B	-0.011000	0.1102	56	-0.100	0.9996
10 - B	0.000000	0.1102	56	0.000	1.0000

Probiotique = PANTOEA SP., Time = 72:

contrast	estimate	SE	df	t.ratio	p.value
0.1 - 1	-0.001667	0.1367	56	-0.012	1.0000
0.1 - 10	-0.219333	0.1367	56	-1.605	0.3843
0.1 - B	-0.084500	0.1367	56	-0.618	0.9258
1 - 10	-0.217667	0.1367	56	-1.593	0.3911
1 - B	-0.082833	0.1367	56	-0.606	0.9297
10 - B	0.134833	0.1367	56	0.987	0.7577

Probiotique = SERRATIA SP.2, Time = 72:

contrast	estimate	SE	df	t.ratio	p.value
0.1 - 1	-0.090000	0.2320	56	-0.388	0.9800
0.1 - 10	-0.087000	0.2320	56	-0.375	0.9818

0.1 - B	-0.033667	0.2320	56	-0.145	0.9989
1 - 10	0.003000	0.2320	56	0.013	1.0000
1 - B	0.056333	0.2320	56	0.243	0.9949
10 - B	0.053333	0.2320	56	0.230	0.9957

Probiotique = HAFNIA SP., Time = 72:					
contrast	estimate	SE	df	t.ratio	p.value
0.1 - 1	-0.016667	0.1160	56	-0.144	0.9989
0.1 - 10	-0.061333	0.1160	56	-0.529	0.9517
0.1 - B	0.513667	0.1160	56	4.430	0.0003
1 - 10	-0.044667	0.1160	56	-0.385	0.9804
1 - B	0.530333	0.1160	56	4.574	0.0002
10 - B	0.575000	0.1160	56	4.959	<.0001

Probiotique = ENTEROBACTER SP., Time = 72:					
contrast	estimate	SE	df	t.ratio	p.value
0.1 - 1	-0.397333	0.1243	56	-3.196	0.0119
0.1 - 10	-0.321333	0.1243	56	-2.585	0.0581
0.1 - B	-0.280667	0.1243	56	-2.258	0.1205
1 - 10	0.076000	0.1243	56	0.611	0.9280
1 - B	0.116667	0.1243	56	0.939	0.7843
10 - B	0.040667	0.1243	56	0.327	0.9878

Table S2: Significant difference in the bacterial growth rate between each pair of probiotic candidates exposed at different treatments: 0.15, 1, 10 ppb and control (no pesticide) at specific time points. Significant p-values < 0.05; p-value adjustment with Tukey's method.

**Trt = 0.15 ppb; Time = 6.**					
Contrast	Estimate	SE	df	t.ratio	p-value
EDWARDSIELLA SP.–RAHNELLA SP.	0.424792	0.099	56	4.290	0.0013
EDWARDSIELLA SP.–PANTOEA SP.	0.557981	0.114	56	4.881	0.0002
EDWARDSIELLA SP.–HAFNIA SP.	0.443528	0.102	56	4.338	0.0011
EDWARDSIELLA SP.–ENTEROBACTER SP.	0.591472	0.107	56	5.526	<.0001
SERRATIA SP.1–PANTOEA SP.	0 .442242	0.131	56	3.372	0.0217
SERRATIA SP.1–ENTEROBACTER SP.	0.475733	0.125	56	3.810	0.0061
**Trt = 1 ppb; Time = 6**					

Contrast	Estimate	SE	df	t.ratio	p-value
EDWARDSIELLA SP.–RAHNELLA SP.	0.371556	0.099	56	3.752	0.0072
EDWARDSIELLA SP.–PANTOEA SP.	0.478569	0.114	56	4.186	0.0019
EDWARDSIELLA SP.–HAFNIA SP.	0.331486	0.102	56	3.242	0.0309
SERRATIA SP.1–RAHNELLA SP.	0.366785	0.118	56	3.107	0.0442
SERRATIA SP.1–PANTOEA SP.	0.473799	0.131	56	3.612	0.0109
SERRATIA SP.1–ENTEROBACTER SP.	0.402827	0.125	56	3.226	0.0323
**Trt = 10 ppb; Time = 6**					
Contrast	Estimate	SE	df	t.ratio	p-value
EDWARDSIELLA SP.–RAHNELLA SP.	0.380069	0.099	56	3.838	0.0056
EDWARDSIELLA SP.–PANTOEA SP.	0.529944	0.114	56	4.635	0.0004
EDWARDSIELLA SP.–HAFNIA SP.	0.342597	0.102	56	3.351	0.0230
EDWARDSIELLA SP.–ENTEROBACTER SP.	0.394361	0.107	56	3.684	0.0088
**Trt = Control; Time = 6**					
Contrast	Estimate	SE	df	t.ratio	p-value
EDWARDSIELLA SP.–RAHNELLA SP.	0.462375	0.099	56	4.670	0.0004
EDWARDSIELLA SP.–PANTOEA SP.	0.636722	0.114	56	5.569	<.0001
EDWARDSIELLA SP.–HAFNIA SP.	0.752458	0.102	56	7.360	<.0001

EDWARDSIELLA SP.–ENTEROBACTER SP.	0.508569	0.107	56	4.752	0.0003
SERRATIA SP.1–PANTOEA SP.	0.421944	0.131	56	3.217.	0.0331
SERRATIA SP.1–HAFNIA SP.	0.537681	0.121	56	4.452	0.0008
SERRATIA SP.2–HAFNIA SP.	0.593444	0.183	56	3.235	0.0315
**Trt = 0.15 ppb; Time = 12**					
Contrast	Estimate	SE	df	t.ratio	p-value
EDWARDSIELLA SP.–RAHNELLA SP.	0.402306	0.099	56	4.063	0.0028
EDWARDSIELLA SP.–PANTOEA SP.	0.713784	0.114	56	6.243	<.0001
EDWARDSIELLA SP.–HAFNIA SP.	0.480042	0.102	56	4.695	0.0003
EDWARDSIELLA SP.–ENTEROBACTER SP.	0.612181	0.107	56	5.720	<.0001
SERRATIA SP.1–PANTOEA SP.	0.669708	0.131	56	5.106	0.0001
SERRATIA SP.1–HAFNIA SP.	0.435966	0.121	56	3.610	0.0110
SERRATIA SP.1–ENTEROBACTER SP.	0.568105	0.125	56	4.550	0.0006
**Trt = 1 ppb; Time = 12**					
Contrast	Estimate	SE	df	t.ratio	p-value
EDWARDSIELLA SP.–RAHNELLA SP.	0.612431	0.099	56	6.185	<.0001
EDWARDSIELLA SP.–PANTOEA SP.	0.716750	0.114	56	6.269	<.0001
EDWARDSIELLA SP.–HAFNIA SP.	0.550292	0.102	56	5.382.	<.0001

EDWARDSIELLA SP.–ENTEROBACTER SP.	0.463319	0.107	56	4.329	0.0012
SERRATIA SP.1–RAHNELLA SP.	0.583625	0.118	56	4.943	0.0001
SERRATIA SP.1–PANTOEA SP.	0.687944	0.131	56	5.245	<.0001
SERRATIA SP.1–HAFNIA SP.	0.521486	0.121	56	4.318	0.0012
SERRATIA SP.1–ENTEROBACTER SP.	0.434514	0.125	56	3.480	0.0160
**Trt = 10 ppb; Time = 12**					
Contrast	Estimate	SE	df	t.ratio	p-value
EDWARDSIELLA SP.–RAHNELLA SP.	0.587042	0.099	56	5.929	<.0001
EDWARDSIELLA SP.–PANTOEA SP.	0.727458	0.114	56	6.363	<.0001
EDWARDSIELLA SP.–HAFNIA SP.	0.561153	0.102	56	5.489	<.0001
EDWARDSIELLA SP.–ENTEROBACTER SP.	0.532056	0.107	56	4.971	0.0001
SERRATIA SP.1–RAHNELLA SP.	0.492722	0.118	56	4.173.	0.0019
SERRATIA SP.1–PANTOEA SP.	0.633139	0.131	56	4.827	0.0002
SERRATIA SP.1–HAFNIA SP.	0.466833	0.121	56	3.865	0.0051
SERRATIA SP.1–ENTEROBACTER SP.	0.437736	0.125	56	3.506	0.0149
**Trt = Control; Time = 12**					
Contrast	Estimate	SE	df	t.ratio	p-value
EDWARDSIELLA SP.–RAHNELLA SP.	0.606306	0.099	56	6.123	<.0001

EDWARDSIELLA SP.–PANTOEA SP.	0.772653	0.114	56	6.758	<.0001
EDWARDSIELLA SP.–HAFNIA SP.	0.936194	0.102	56	9.157	<.0001
EDWARDSIELLA SP.–ENTEROBACTER SP.	0.496681	0.107	56	4.640	0.0004
SERRATIA SP.1–RAHNELLA SP.	0.518514	0.118	56	4.392	0.0009
SERRATIA SP.1–PANTOEA SP.	0.684861	0.131	56	5.221	0.0001
SERRATIA SP.1–HAFNIA SP.	0.848403	0.121	56	7.024	<.0001
SERRATIA SP.1–ENTEROBACTER SP.	0.408889	0.125	56	3.275	0.0283
HAFNIA SP.–ENTEROBACTER SP.	-0.439514	0.120	56	-3.656.	0.0096
**Trt = 0.15 ppb; Time = 18**					
Contrast	Estimate	SE	df	t.ratio	p-value
EDWARDSIELLA SP.–RAHNELLA SP.	0.371583	0.099	56	3.753	0.0072
EDWARDSIELLA SP.–PANTOEA SP.	0.857428	0.114	56	7.500.	<.0001
EDWARDSIELLA SP.–HAFNIA SP.	0.440806	0.102	56	4.311.	0.0012
EDWARDSIELLA SP.–ENTEROBACTER SP.	0.649319	0.107	56	6.067	<.0001
SERRATIA SP.1–PANTOEA SP.	0.801888	0.131	56	6.113	<.0001
SERRATIA SP.1–HAFNIA SP.	0.385266	0.121	56	3.190	0.0355
SERRATIA SP.1–ENTEROBACTER SP.	0.593780	0.125	56	4.755	0.0003
RAHNELLA SP.–PANTOEA SP.	0.485845	0.124	56	3.913	0.0044
PANTOEA SP.–HAFNIA SP.	-0.416623	0.127	56	-3.287.	0.0274

**Trt = 1 ppb; Time = 18**

Contrast	Estimate	SE	df	t.ratio	p-value
EDWARDSIELLA SP.–RAHNELLA SP.	0.459819	0.099	56	4.644	0.0004
EDWARDSIELLA SP.–PANTOEA SP.	0.802833	0.114	56	7.022	<.0001
EDWARDSIELLA SP.–HAFNIA SP.	0.519778	0.102	56	5.084	0.0001
EDWARDSIELLA SP.–ENTEROBACTER SP.	0.330903	0.107	56	3.092	0.0459
SERRATIA SP.1–RAHNELLA SP.	0.552898	0.118	56	4.683	0.0004
SERRATIA SP.1–PANTOEA SP.	0.895912	0.131	56	6.830	<.0001
SERRATIA SP.1–HAFNIA SP.	0.612856	0.121	56	5.074	0.0001
SERRATIA SP.1–ENTEROBACTER SP.	0.423981	0.125	56	3.396.	0.0203
PANTOEA SP.–ENTEROBACTER SP.	-0.471931	0.131	56	-3.612.	0.0109

**Trt = 10 ppb; Time = 18**

Contrast	Estimate	SE	df	t.ratio	p-value
EDWARDSIELLA SP.–RAHNELLA SP.	0.487764	0.099	56	4.926	0.0002
EDWARDSIELLA SP.–PANTOEA SP.	0.737833	0.114	56	6.454	<.0001
EDWARDSIELLA SP.–HAFNIA SP.	0.519444	0.102	56	5.081	0.0001
EDWARDSIELLA SP.–ENTEROBACTER SP.	0.372875	0.107	56	3.484.	0.0159
SERRATIA SP.1–RAHNELLA SP.	0.568597	0.118	56	4.816	0.0002

SERRATIA SP.1–PANTOEA SP.	0.818667	0.131	56	6.241	<.0001
SERRATIA SP.1–HAFNIA SP.	0.600278	0.121	56	4.970	0.0001
SERRATIA SP.1–ENTEROBACTER SP.	0.453708	0.125	56	3.634.	0.0103
**Trt = Control; Time = 18**					
Contrast	Estimate	SE	df	t.ratio	p-value
EDWARDSIELLA SP.–RAHNELLA SP.	0.527681	0.099	56	5.329	<.0001
EDWARDSIELLA SP.–PANTOEA SP.	0.807764	0.114	56	7.065.	<.0001
EDWARDSIELLA SP.–HAFNIA SP.	0.967944	0.102	56	9.467	<.0001
EDWARDSIELLA SP.–ENTEROBACTER SP.	0.371875	0.107	56	3.474.	0.0163
SERRATIA SP.1–RAHNELLA SP.	0.588625	0.118	56	4.986	0.0001
SERRATIA SP.1–PANTOEA SP.	0.868708	0.131	56	6.623	<.0001
SERRATIA SP.1–HAFNIA SP.	1.028889	0.121	56	8.519	<.0001
SERRATIA SP.1–ENTEROBACTER SP.	0.432819	0.125	56	3.466.	0.0167
RAHNELLA SP.–HAFNIA SP.	0.440264	0.113	56	3.892	0.0047
PANTOEA SP.–ENTEROBACTER SP.	-0.435889	0.131	56	-3.337.	0.0239
SERRATIA SP.2–HAFNIA SP.	0.711750	0.183	56	3.880.	0.0049
HAFNIA SP.–ENTEROBACTER SP.	-0.596069	0.120	56	-4.959	0.0001
**Trt = 0.15 ppb; Time = 24**					

Contrast	Estimate	SE	df	t.ratio	p-value
EDWARDSIELLA SP.–RAHNELLA SP.	0.392569	0.099	56	3.965	0.0038
EDWARDSIELLA SP.–PANTOEA SP.	0.892397	0.114	56	7.806.	<.0001
EDWARDSIELLA SP.–HAFNIA SP.	0.433361	0.102	56	4.239.	0.0016
EDWARDSIELLA SP.–ENTEROBACTER SP.	0.678278	0.107	56	6.337	<.0001
SERRATIA SP.1–PANTOEA SP.	0.834202	0.131	56	6.360	<.0001
SERRATIA SP.1–HAFNIA SP.	0.375165	0.121	56	3.106	0.0442
SERRATIA SP.1–ENTEROBACTER SP.	0.620082	0.125	56	4.966	0.0001
RAHNELLA SP.–PANTOEA SP.	0.499828	0.124	56	4.026	0.0031
PANTOEA SP.–HAFNIA SP.	-0.459036	0.127	56	-3.622	0.0106
**Trt = 1 ppb; Time = 24**					
Contrast	Estimate	SE	df	t.ratio	p-value
EDWARDSIELLA SP.–RAHNELLA SP.	0.412764	0.099	56	4.169	0.0020
EDWARDSIELLA SP.–PANTOEA SP.	0.848722	0.114	56	7.424	<.0001
EDWARDSIELLA SP.–HAFNIA SP.	0.510972	0.102	56	4.998	0.0001
SERRATIA SP.1–RAHNELLA SP.	0.489553	0.118	56	4.147	0.0021
SERRATIA SP.1–PANTOEA SP.	0.925512	0.131	56	7.056.	<.0001
SERRATIA SP.1–HAFNIA SP.	0.587762	0.121	56	4.866	0.0002
SERRATIA SP.1–ENTEROBACTER SP.	0.402442	0.125	56	3.223	0.0325

Contrast	Estimate	SE	df	t.ratio	p-value
RAHNELLA SP.–ANTOEA SP.	0.435958	0.124	56	3.511	0.0146
PANTOEA SP.–ENTEROBACTER SP.	-0.523069	0.131	56	-4.004.	0.0033

Trt = 10 ppb; Time = 24

Contrast	Estimate	SE	df	t.ratio	p-value
EDWARDSIELLA SP.–RAHNELLA SP.	0.383153	0.099	56	3.870	0.0050
EDWARDSIELLA SP.–PANTOEA SP.	0.744486	0.114	56	6.512.	<.0001
EDWARDSIELLA SP.–HAFNIA SP.	0.516431	0.102	56	5.051	0.0001
SERRATIA SP.1–RAHNELLA SP.	0.464681	0.118	56	3.936	0.0041
SERRATIA SP.1–PANTOEA SP.	0.826014	0.131	56	6.297	<.0001
SERRATIA SP.1–HAFNIA SP.	0.597958	0.121	56	4.951	0.0001
SERRATIA SP.1–ENTEROBACTER SP.	0.390236	0.125	56	3.125.	0.0421
PANTOEA SP.–ENTEROBACTER SP.	-0.435778	0.131	56	-3.336.	0.0240

Trt = Control; Time = 24

Contrast	Estimate	SE	df	t.ratio	p-value
EDWARDSIELLA SP.–RAHNELLA SP.	0.398236	0.099	56	4.022	0.0031
EDWARDSIELLA SP.–PANTOEA SP.	0.842056	0.114	56	7.365	<.0001
EDWARDSIELLA SP.–HAFNIA SP.	0.985486	0.102	56	9.639	<.0001
EDWARDSIELLA SP.–ENTEROBACTER SP.	0.337611	0.107	56	3.154	0.0390

SERRATIA SP.1–RAHNELLA SP.	0.503653	0.118	56	4.266.	0.0014
SERRATIA SP.1–PANTOEA SP.	0.947472	0.131	56	7.223	<.0001
SERRATIA SP.1–HAFNIA SP.	1.090903	0.121	56	9.032	<.0001
SERRATIA SP.1–ENTEROBACTER SP.	0.443028	0.125	56	3.548	0.0132
RAHNELLA SP.–PANTOEA SP.	0.443819	0.124	56	3.575	0.0122
RAHNELLA SP.–HAFNIA SP.	0.587250	0.113	56	5.191	0.0001
PANTOEA SP.–ENTEROBACTER SP.	-0.504444	0.131	56	-3.861.	0.0052
SERRATIA SP.2–HAFNIA SP.	0.680556	0.183	56	3.710	0.0082
HAFNIA SP.–ENTEROBACTER SP.	-0.647875	0.120	56	-5.390	<.0001
**Trt = 0.15 ppb; Time = 30**					
Contrast	Estimate	SE	df	t.ratio	p-value
EDWARDSIELLA SP.–RAHNELLA SP.	0.382278	0.099	56	3.861	0.0052
EDWARDSIELLA SP.–PANTOEA SP.	0.889891	0.114	56	7.784	<.0001
EDWARDSIELLA SP.–HAFNIA SP.	0.417639	0.102	56	4.085	0.0026
SERRATIA SP.1–PANTOEA SP.	0.836651	0.131	56	6.378.	<.0001
SERRATIA SP.1–ENTEROBACTER SP.	0.607538	0.125	56	4.866.	0.0002
RAHNELLA SP.–PANTOEA SP.	0.507614	0.124	56	4.089	0.0025
PANTOEA SP.–HAFNIA SP.	-0.472252	0.127	56	-3.726	0.0078

**Trt = 1 ppb; Time = 30**					
Contrast	Estimate	SE	df	t.ratio	p-value
EDWARDSIELLA SP.– RAHNELLA SP.	0.365569	0.099	56	3.692	0.0086
EDWARDSIELLA SP.–PANTOEA SP.	0.872597	0.114	56	7.632	<.0001
EDWARDSIELLA SP.–HAFNIA SP.	0.446917	0.102	56	4.371	0.0010
SERRATIA SP.1–RAHNELLA SP.	0.418400	0.118	56	3.544	0.0133
SERRATIA SP.1–PANTOEA SP.	0.925427	0.131	56	7.055	<.0001
SERRATIA SP.1–HAFNIA SP.	0.499747	0.121	56	4.138	0.0022
RAHNELLA SP.–PANTOEA SP.	0.507028	0.124	56	4.084	0.0026
PANTOEA SP.–HAFNIA SP.	-0.425681	0.127	56	-3.359	0.0225
PANTOEA SP.–ENTEROBACTER SP.	-0.570347	0.131	56	-4.366	0.0010
**Trt = 10 ppb; Time = 30**					
Contrast	Estimate	SE	df	t.ratio	p-value
EDWARDSIELLA SP.–RAHNELLA SP.	0.327347	0.099	56	3.306	0.0260
EDWARDSIELLA SP.–PANTOEA SP.	0.690833	0.114	56	6.043	<.0001
EDWARDSIELLA SP.–HAFNIA SP.	0.505514	0.102	56	4.944	0.0001
SERRATIA SP.1–RAHNELLA SP.	0.383236	0.118	56	3.246	0.0306
SERRATIA SP.1–PANTOEA SP.	0.746722	0.131	56	5.693.	<.0001
SERRATIA SP.1–HAFNIA SP.	0.561403	0.121	56	4.648	0.0004

Contrast	Estimate	SE	df	t.ratio	p-value
PANTOEA SP.–ENTEROBACTER SP.	-0.401250	0.131	56	-3.071	0.0483
**Trt = Control; Time = 30**					
Contrast	Estimate	SE	df	t.ratio	p-value
EDWARDSIELLA SP.–PANTOEA SP.	0.810736	0.114	56	7.091	<.0001
EDWARDSIELLA SP.–HAFNIA SP.	0.967000	0.102	56	9.458	<.0001
SERRATIA SP.1–RAHNELLA SP.	0.433014	0.118	56	3.668	0.0093
SERRATIA SP.1–PANTOEA SP.	0.941278	0.131	56	7.176	<.0001
SERRATIA SP.1–HAFNIA SP.	1.097542	0.121	56	9.087	<.0001
SERRATIA SP.1–ENTEROBACTER SP.	0.420889	0.125	56	3.371	0.0218
RAHNELLA SP.–PANTOEA SP.	0.508264	0.124	56	4.094	0.0025
RAHNELLA SP.–HAFNIA SP.	0.664528	0.113	56	5.874	<.0001
PANTOEA SP.–ENTEROBACTER SP.	-0.520389	0.131	56	-3.983.	0.0035
SERRATIA SP.2–HAFNIA SP.	0.633861	0.183	56	3.456	0.0172
HAFNIA SP.–ENTEROBACTER SP.	-0.676653	0.120	56	-5.629	<.0001
**Trt = 0.15 ppb; Time = 36;**					
Contrast	Estimate	SE	df	t.ratio	p-value
EDWARDSIELLA SP.–RAHNELLA SP.	0.355472	0.099	56	3.590	0.0117
EDWARDSIELLA SP.–PANTOEA SP.	0.845495	0.114	56	7.395	<.0001

EDWARDSIELLA SP.–HAFNIA SP.	0.397903	0.102	56	3.892	0.0047
EDWARDSIELLA SP.–ENTEROBACTER SP.	0.621292	0.107	56	5.805	<.0001
SERRATIA SP.1–PANTOEA SP.	0.830298	0.131	56	6.330	<.0001
SERRATIA SP.1–HAFNIA SP.	0.382707	0.121	56	3.169.	0.0376
SERRATIA SP.1–ENTEROBACTER SP.	0.606096	0.125	56	4.854	0.0002
RAHNELLA SP.–PANTOEA SP.	0.490022	0.124	56	3.947	0.0040
PANTOEA SP.–HAFNIA SP.	-0.447592	0.127	56	-3.532	0.0138
**Trt = 1 ppb; Time = 36**					
Contrast	Estimate	SE	df	t.ratio	p-value
EDWARDSIELLA SP.– RAHNELLA SP.	0.328153	0.099	56	3.314	0.0255
EDWARDSIELLA SP.–PANTOEA SP.	0.856278	0.114	56	7.490	<.0001
EDWARDSIELLA SP.–HAFNIA SP.	0.369292	0.102	56	3.612	0.0109
SERRATIA SP.1–PANTOEA SP.	0.838629	0.131	56	6.393	<.0001
RAHNELLA SP.–PANTOEA SP.	0.528125	0.124	56	4.254	0.0015
PANTOEA SP.–HAFNIA SP.	-0.486986	0.127	56	-3.842	0.0055
PANTOEA SP.–ENTEROBACTER SP.	-0.602069	0.131	56	-4.609	0.0005
**Trt = 10 ppb; Time = 36**					
Contrast	Estimate	SE	df	t.ratio	p-value

EDWARDSIELLA SP.–PANTOEA SP.	0.625403	0.114	56	5.470	<.0001
EDWARDSIELLA SP.–HAFNIA SP.	0.427028	0.102	56	4.177	0.0019
SERRATIA SP.1–PANTOEA SP.	0.623528	0.131	56	4.754	0.0003
SERRATIA SP.1–HAFNIA SP.	0.425153	0.121	56	3.520	0.0143
**Trt = Control; Time = 36**					
Contrast	Estimate	SE	df	t.ratio	p-value
EDWARDSIELLA SP.–PANTOEA SP.	0.728639	0.114	56	6.373	<.0001
EDWARDSIELLA SP.–HAFNIA SP.	0.893250	0.102	56	8.737	<.0001
SERRATIA SP.1–PANTOEA SP.	0.822514	0.131	56	6.271	<.0001
SERRATIA SP.1–HAFNIA SP.	0.987125	0.121	56	8.173	<.0001
RAHNELLA SP.–PANTOEA SP.	0.506681	0.124	56	4.081	0.0026
RAHNELLA SP.–HAFNIA SP.	0.671292	0.113	56	5.934	<.0001
PANTOEA SP.–ENTEROBACTER SP.	-0.512403	0.131	56	-3.922	0.0043
HAFNIA SP.–ENTEROBACTER SP.	-0.677014	0.120	56	-5.632.	<.0001
**Trt = 0.15 ppb; Time = 42**					
Contrast	Estimate	SE	df	t.ratio	p-value
EDWARDSIELLA SP.–PANTOEA SP.	0.731359	0.114	56	6.397	<.0001
EDWARDSIELLA SP.–HAFNIA SP.	0.332486	0.102	56	3.252	0.0301

EDWARDSIELLA SP.–ENTEROBACTER SP.	0.515750	0.107	56	4.819	0.0002
SERRATIA SP.1–PANTOEA SP.	0.756456	0.131	56	5.767	<.0001
SERRATIA SP.1–ENTEROBACTER SP.	0.540848	0.125	56	4.332	0.0012
RAHNELLA SP.–PANTOEA SP.	0.456984	0.124	56	3.681	0.0089
PANTOEA SP.–HAFNIA SP.	-0.398873	0.127	56	-3.147	0.0398
**Trt = 1 ppb; Time = 42**					
Contrast	Estimate	SE	df	t.ratio	p-value
EDWARDSIELLA SP.–PANTOEA SP.	0.757958	0.114	56	6.630	<.0001
SERRATIA SP.1–PANTOEA SP.	0.723709	0.131	56	5.517	<.0001
RAHNELLA SP.–PANTOEA SP.	0.515417	0.124	56	4.151.	0.0021
PANTOEA SP.–HAFNIA SP.	-0.470167	0.127	56	-3.710	0.0082
PANTOEA SP.–ENTEROBACTER SP.	-0.600528	0.131	56	-4.597	0.0005
**Trt = 10 ppb; Time = 42**					
Contrast	Estimate	SE	df	t.ratio	p-value
EDWARDSIELLA SP.–PANTOEA SP.	0.509861	0.114	56	4.460	0.0008
SERRATIA SP.1–PANTOEA SP.	0.524694	0.131	56	4.000	0.0034
**Trt = Control; Time = 42**					

Contrast	Estimate	SE	df	t.ratio	pvalue
EDWARDSIELLA SP.–PANTOEA SP.	0.606014	0.114	56	5.301	<.0001
EDWARDSIELLA SP.–HAFNIA SP.	0.778472	0.102	56	7.614.	<.0001
SERRATIA SP.1–HAFNIA SP.	0.894653	0.121	56	7.407	<.0001
RAHNELLA SP.–PANTOEA SP.	0.487083	0.124	56	3.923	0.0043
RAHNELLA SP.–HAFNIA SP.	0.659542	0.113	56	5.830	<.0001
PANTOEA SP.–ENTEROBACTER SP.	-0.488972	0.131	56	-3.743	0.0074
HAFNIA SP.–ENTEROBACTER SP.	-0.661431	0.120	56	-5.503	<.0001
**Trt = 0.15 ppb; Time = 48**					
Contrast	Estimate	SE	df	t.ratio	p-value
EDWARDSIELLA SP.–PANTOEA SP.	0.634332	0.114	56	5.548	<.0001
EDWARDSIELLA SP.–ENTEROBACTER SP.	0.431958	0.107	56	4.036	0.0030
SERRATIA SP.1–PANTOEA SP.	0.635394	0.131	56	4.844	0.0002
SERRATIA SP.1–ENTEROBACTER SP.	0.433020	0.125	56	3.468	0.0166
RAHNELLA SP.–PANTOEA SP.	0.413540	0.124	56	3.331	0.0243
**Trt = 1 ppb; Time = 48**					
Contrast	Estimate	SE	df	t.ratio	p-value
EDWARDSIELLA SP.–PANTOEA SP.	0.635083	0.114	56	5.555	<.0001

Contrast	Estimate	SE	df	t.ratio	p-value
SERRATIA SP.1–PANTOEA SP.	0.639553	0.131	56	4.876	0.0002
RAHNELLA SP.–PANTOEA SP.	0.487681	0.124	56	3.928	0.0042
PANTOEA SP.–HAFNIA SP.	-0.440097	0.127	56	-3.472.	0.0164
PANTOEA SP.–ENTEROBACTER SP.	-0.585861	0.131	56	-4.485	0.0007
**Trt = 10 ppb; Time = 48**					
Contrast	Estimate	SE	df	t.ratio	p-value
EDWARDSIELLA SP.–PANTOEA SP.	0.410708	0.114	56	3.592	0.0116
SERRATIA SP.1–PANTOEA SP.	0.457417	0.131	56	3.487	0.0157
**Trt = Control; Time = 48**					
Contrast	Estimate	SE	df	t.ratio	p-value
EDWARDSIELLA SP.–PANTOEA SP.	0.538097	0.114	56	4.707	0.0003
EDWARDSIELLA SP.–HAFNIA SP.	0.711306	0.102	56	6.957	<.0001
SERRATIA SP.1–PANTOEA SP.	0.641611	0.131	56	4.891	0.0002
SERRATIA SP.1–HAFNIA SP.	0.814819	0.121	56	6.746	<.0001
RAHNELLA SP.–HAFNIA SP.	0.613736	0.113	56	5.425	<.0001
PANTOEA SP.–ENTEROBACTER SP.	-0.458667	0.131	56	-3.511	0.0147
HAFNIA SP.–ENTEROBACTER SP.	-0.631875	0.120	56	-5.257	<.0001

Trt = 0.15 ppb; Time = 54					
Contrast	Estimate	SE	df	t.ratio	p-value
EDWARDSIELLA SP.–ENTEROBACTER SP.	0.397875	0.107	56	3.717	0.0080
SERRATIA SP.1–PANTOEA SP.	0.533338	0.131	56	4.066	0.0027
RAHNELLA SP.–PANTOEA SP.	0.433411	0.124	56	3.491	0.0155
PANTOEA SP.–HAFNIA SP.	-0.413342	0.127	56	-3.261.	0.0294
**Trt = 1 ppb; Time = 54**					
Contrast	Estimate	SE	df	t.ratio	p-value
EDWARDSIELLA SP.–PANTOEA SP.	0.581042	0.114	56	5.082	0.0001
SERRATIA SP.1–PANTOEA SP.	0.541887	0.131	56	4.131	0.0022
RAHNELLA SP.–PANTOEA SP.	0.463625	0.124	56	3.734	0.0076
PANTOEA SP.–HAFNIA SP.	-0.436611	0.127	56	-3.445	0.0177
PANTOEA SP.–ENTEROBACTER SP.	-0.570833	0.131	56	-4.370	0.0010
**Trt = 10 ppb; Time = 54**					
Contrast	Estimate	SE	df	t.ratio	p-value
EDWARDSIELLA SP.–PANTOEA SP.	0.368028	0.114	56	3.219	0.0329
**Trt = Control; Time = 54**					

Contrast	Estimate	SE	df	t.ratio	p-value
EDWARDSIELLA SP.–PANTOEA SP.	0.514889	0.114	56	4.504	0.0007
EDWARDSIELLA SP.–HAFNIA SP.	0.676431	0.102	56	6.616	<.0001
SERRATIA SP.1–PANTOEA SP.	0.547333	0.131	56	4.173	0.0019
SERRATIA SP.1–HAFNIA SP.	0.708875	0.121	56	5.869	<.0001
RAHNELLA SP.–PANTOEA SP.	0.422403	0.124	56	3.402	0.0200
RAHNELLA SP.–HAFNIA SP.	0.583944	0.113	56	5.162	0.0001
PANTOEA SP.–ENTEROBACTER SP. C	-0.436417	0.131	56	-3.341	0.0237
HAFNIA SP.–ENTEROBACTER SP.	-0.597958	0.120	56	-4.975	0.0001
**Trt = 0.15 ppb; Time = 60**					
Contrast	Estimate	SE	df	t.ratio	p-value
EDWARDSIELLA SP.–PANTOEA SP.	0.597995	0.114	56	5.231	0.0001
EDWARDSIELLA SP.–ENTEROBACTER SP.	0.369375	0.107	56	3.451	0.0174
SERRATIA SP.1–PANTOEA SP.	0.488401	0.131	56	3.723	0.0079
RAHNELLA SP.–PANTOEA SP.	0.465537	0.124	56	3.750	0.0073
PANTOEA SP.–HAFNIA SP.	-0.446370	0.127	56	-3.522	0.0142
**Trt = 1 ppb; Time = 60**					
Contrast	Estimate	SE	df	t.ratio	p-value

EDWARDSIELLA SP.–PANTOEA SP.	0.577042	0.114	56	5.047	0.0001
RAHNELLA SP.–PANTOEA SP.	0.487028	0.124	56	3.923	0.0043
PANTOEA SP.–HAFNIA SP.	-0.459264	0.127	56	-3.624	0.0106
PANTOEA SP.–ENTEROBACTER SP.	-0.575944	0.131	56	-4.409	0.0009
**Trt = 10 ppb; Time = 60**					
Contrast	Estimate	SE	df	t.ratio	p-value
EDWARDSIELLA SP.–PANTOEA SP.	0.352403	0.114	56	3.082	0.0470
**Trt = Control; Time = 60**					
Contrast	Estimate	SE	df	t.ratio	p-value
EDWARDSIELLA SP.–PANTOEA SP.	0.503611	0.114	56	4.405	0.0009
EDWARDSIELLA SP.–HAFNIA SP.	0.644931	0.102	56	6.308	<.0001
SERRATIA SP.1–PANTOEA SP.	0.459361	0.131	56	3.502	0.0150
SERRATIA SP.1–HAFNIA SP.	0.600681	0.121	56	4.973	0.0001
RAHNELLA SP.–PANTOEA SP.	0.437069	0.124	56	3.520	0.0143
RAHNELLA SP.–HAFNIA SP.	0.578389	0.113	56	5.113	0.0001
PANTOEA SP.–ENTEROBACTER SP.	-0.437819	0.131	56	-3.351	0.0230
HAFNIA SP.–ENTEROBACTER SP.	-0.579139	0.120	56	-4.818	0.0002

Trt = 0.15 ppb; Time = 66

Contrast	Estimate	SE	df	t.ratio	p-value
EDWARDSIELLA SP.–PANTOEA SP.	0.592735	0.114	56	5.185	0.0001
EDWARDSIELLA SP.–ENTEROBACTER SP.	0.328486	0.107	56	3.069	0.0486
SERRATIA SP.1–PANTOEA SP.	0.467298	0.131	56	3.563	0.0126
RAHNELLA SP.–PANTOEA SP.	0.471804	0.124	56	3.800	0.0062
PANTOEA SP.–HAFNIA SP.	-0.467263	0.127	56	-3.687	0.0088

Trt = 1 ppb; Time = 66

Contrast	Estimate	SE	df	t.ratio	p-value
EDWARDSIELLA SP.–PANTOEA SP.	0.581181	0.114	56	5.083	0.0001
SERRATIA SP.1–PANTOEA SP.	0.438363	0.131	56	3.342	0.0236
RAHNELLA SP.–PANTOEA SP.	0.507056	0.124	56	4.084	0.0026
PANTOEA SP.–HAFNIA SP.	-0.481375	0.127	56	-3.798	0.0063
PANTOEA SP.–ENTEROBACTER SP.	-0.639139	0.131	56	-4.892	0.0002

Trt = Control; Time = 66

Contrast	Estimate	SE	df	t.ratio	p-value
EDWARDSIELLA SP.–PANTOEA SP.	0.496333	0.114	56	4.341	0.0011
EDWARDSIELLA SP.–HAFNIA SP.	0.629347	0.102	56	6.156	<.0001

SERRATIA SP.1–HAFNIA SP.	0.527597	0.121	56	4.368	0.0010
RAHNELLA SP.–PANTOEA SP.	0.433056	0.124	56	3.488	0.0157
RAHNELLA SP.–HAFNIA SP.	0.566069	0.113	56	5.004	0.0001
PANTOEA SP.–ENTEROBACTER SP.	-0.460833	0.131	56	-3.528	0.0140
HAFNIA SP.–ENTEROBACTER SP.	-0.593847	0.120	56	-4.940	0.0001
**Trt = 0.15 ppb; Time = 72**					
Contrast	Estimate	SE	df	t.ratio	p-value
EDWARDSIELLA SP.–PANTOEA SP.	0.583333	0.114	56	5.102	0.0001
SERRATIA SP.1–PANTOEA SP.	0.452333	0.131	56	3.448	0.0175
RAHNELLA SP.–PANTOEA SP.	0.470667	0.124	56	3.791	0.0064
PANTOEA SP.–HAFNIA SP.	-0.464667	0.127	56	-3.666	0.0093
**Trt = 1 ppb; Time = 72**					
Contrast	Estimate	SE	df	t.ratio	p-value
EDWARDSIELLA SP.–PANTOEA SP.	0.576333	0.114	56	5.041	0.0001
SERRATIA SP.1–PANTOEA SP.	0.421667	0.131	56	3.215	0.0333
RAHNELLA SP.–PANTOEA SP.	0.494333	0.124	56	3.982	0.0036
PANTOEA SP.–HAFNIA SP.	-0.479667	0.127	56	-3.785	0.0065
PANTOEA SP.–ENTEROBACTER SP.	-0.666667	0.131	56	-5.103	0.0001

**Trt = Control; Time = 72**

Contrast	Estimate	SE	df	t.ratio	p-value
EDWARDSIELLA SP.–PANTOEA SP.	0.488833	0.114	56	4.276	0.0014
EDWARDSIELLA SP.–HAFNIA SP.	0.622333.	0.102	56	6.087	<.0001
SERRATIA SP.1–HAFNIA SP.	0.506333	0.121	56	4.192	0.0018
RAHNELLA SP.–PANTOEA SP.	0.422500	0.124	56	3.403	0.0199
RAHNELLA SP.–HAFNIA SP.	0.556000	0.113	56	4.915	0.0002
PANTOEA SP.–ENTEROBACTER SP.	-0.467167	0.131	56	-3.576	0.0122
HAFNIA SP.–ENTEROBACTER SP.	-0.600667	0.120	56	-4.997	0.0001

Table S3: Multiple comparisons of clothianidin degradation using an ANOVA analysis between each pair of probiotic candidates (PC) and with the control (TSB + 0.15 ppb clothianidin) at T24.

	emmean	SE	df	lower.CL	upper.CL	group
Edwardsiella sp.	0.06167	0.02595	16	0.006651	0.1167	a
Serratia sp.1	0.14633	0.02595	16	0.091318	0.2013	a
Serratia sp.2	0.07833	0.02595	16	0.023318	0.1333	a
Rahnella sp.	0.06033	0.02595	16	0.005318	0.1153	a
Pantoea sp.	0.07700	0.02595	16	0.021984	0.1320	a
Hafnia sp.	0.07767	0.02595	16	0.022651	0.1327	a
Enterobacter sp.	0.09700	0.02595	16	0.041984	0.1520	a
Control	0.15300	0.02595	23	0.099314	0.2067	a

Table S4: Multiple comparisons of clothianidin degradation using an ANOVA analysis between each probiotic candidate with the control (TSB + 0.15 ppb Clothianidin) at T48.

Comparisons Control *vs.*	estimate	SE	df	t ratio	p-value
Edwardsiella sp.	0.145667	0.0231	16	6.302	0.0002 ***
Serratia sp.1	0.143000	0.0231	16	6.187	0.0003***
Serratia sp.2	0.131667	0.0231	16	5.696	0.0007***
Rahnella sp.	0.138333	0.0231	16	5.985	0.0004***
Pantoea sp.	0.124000	0.0231	16	5.365	0.0013**
Hafnia sp.	0.116333	0.0231	16	5.033	0.0024**
Enterobacter sp.	0.102000	0.0231	16	4.413	0.0080**

Significant p-values < 0.05; p-value adjustment with Tukey's method.

Table S5: Multiple comparisons of clothianidin degradation using an ANOVA analysis between each probiotic candidate with the control (TSB + 0.15 ppb Clothianidin) at T72.

Comparisons Control *vs.*	estimate	SE	df	t ratio	p-value
Edwardsiella sp.	0.193333	0.0112	16	17.267	< 0.0001
Serratia sp.1	0.192000	0.0112	16	17.148	< 0.0001
Serratia sp.2	0.194667	0.0112	16	17.387	< 0.0001
Rahnella sp.	0.200667	0.0112	16	17.922	< 0.0001
Pantoea sp.	0.200667	0.0112	16	17.922	< 0.0001
Hafnia sp.	0.200667	0.0112	16	17.922	< 0.0001
Enterobacter sp.	0.200667	0.0112	16	17.922	< 0.0001

Significant p-values < 0.05; p-value adjustment with Tukey's method.
